# Deregulation of the Kallikrein Protease Family in the Salivary Glands of the Sjögren’s Syndrome ERdj5 Knockout Mouse Model

**DOI:** 10.3389/fimmu.2021.693911

**Published:** 2021-07-07

**Authors:** Petros Moustardas, Naomi Yamada-Fowler, Eirini Apostolou, Athanasios G. Tzioufas, Maria V. Turkina, Giannis Spyrou

**Affiliations:** ^1^ Department of Biomedical and Clinical Sciences, Faculty of Medicine and Health Sciences, Linköping University, Linköping, Sweden; ^2^ Center for Clinical, Experimental Surgery & Translational Research, Biomedical Research Foundation of the Academy of Athens, Athens, Greece; ^3^ Department of Pathophysiology, School of Medicine, National and Kapodistrian University of Athens, Athens, Greece

**Keywords:** Kallikrein, Sjögren’s syndrome, NGF, proteomic, salivary gland, dimorphism, mouse, autoimmunity

## Abstract

**Introduction:**

The purpose of this study was to identify differentially expressed proteins in salivary glands of the ERdj5 knockout mouse model for Sjögren’s syndrome and to elucidate possible mechanisms for the morbid phenotype development. At the same time, we describe for the first time the sexual dimorphism of the murine submandibular salivary gland at the proteome level.

**Methods:**

We performed Liquid Chromatography/Mass Spectrometry in salivary gland tissues from both sexes of ERdj5 knockout and 129SV wildtype mice. The resulting list of proteins was evaluated with bioinformatic analysis and selected proteins were validated by western blot and immunohistochemistry and further analyzed at the transcription level by qRT-PCR.

**Results:**

We identified 88 deregulated proteins in females, and 55 in males in wildtype *vs* knockout comparisons. In both sexes, Kallikrein 1b22 was highly upregulated (fold change>25, ANOVA p<0.0001), while all other proteases of this family were either downregulated or not significantly affected by the genotype. Bioinformatic analysis revealed a possible connection with the downregulated NGF that was further validated by independent methods. Concurrently, we identified 416 proteins that were significantly different in the salivary gland proteome of wildtype female *vs* male mice and highlighted pathways that could be driving the strong female bias of the pathology.

**Conclusion:**

Our research provides a list of novel targets and supports the involvement of an NGF-mediating proteolytic deregulation pathway as a focus point towards the better understanding of the underlying mechanism of Sjögren’s syndrome.

## Introduction

Sjögren’s syndrome (SS) is a chronic autoimmune disease that is characterised by monocellular lymphocytic infiltration in secretory tissues, such as the salivary (SG) and lachrymal (LG) glands, which results in reduced secretion of tears and saliva and has a potential for malignant lymphoma development (1, 2). Epithelial cells are considered to be conductors of the immune response in SS (3). Over the last years there have been increasing evidence that in the Endoplasmic Reticulum (ER) of epithelial cells, the disturbance of the protein folding process (ER-stress), which leads to the activation of the Unfolded Protein Response (UPR), plays an important role in the initiation and/or perpetuation of autoimmune responses (4) and has been implicated with SS (5–7).

Our recently established ER-stress related Sjögren’s syndrome animal model of ERdj5 knockout in mice (ERdj5^-/-^) also strengthens this connection: ERdj5 is a chaperone protein involved in the ER-associated protein degradation (ERAD) pathway and its removal in mice results in the development of pathological characteristics of SS, like salivary gland inflammatory infiltrations, anti-SSA/Ro and anti-SSB/La autoantibodies, xerostomia and a marked predilection towards female individuals (8). ER-stress and an activated UPR signaling are also prevalent within the salivary glands of both the ERdj5^-/-^ mouse model (9) and in human patients (8).

Inadequate UPR and protein misfolding may contribute to autoimmunity through four possible mechanisms: Recognition of misfolded proteins by immune cells, release of neo-autoantigens by cells that are dying from unrecoverable ER-stress, perturbation of immune-tolerance mechanisms and conferring of a survival advantage to auto­reactive cells by upregulating ERAD proteins (10). The ERdj5^-/-^ mouse model has allowed us to explore more specifically these possibilities and elicit plausible mechanisms of the SS-like phenotype in ERdj5^-/-^ mice. Two major categories of identified proteins found through this research offer a compelling model that is explored in this study: The glandular kallikrein family of serine proteases and the nerve growth factor (NGF), which is a substrate of kallikreins.

Kallikreins (KLK) are a family of serine proteases that were first described for their ability to process kininogens to bradykinin and regulate vasodilation/constriction. Two distinct groups of this family were later identified, the plasma and the glandular kallikreins. In mice, a rich subfamily of the kallikrein 1-related proteins -Klk1b(x)s- is phylogenetically closer to the human glandular KLKs 1-3, containing an ortholog for the human KLK1 (the mouse Klk1, also named mGK6, Klk-6 or Klk1b6), and 13 other klk1b(x)s that do not have orthologs in humans (11). Of those proteases, some retain the specificity to cleave Met-Lys and Arg-Ser bonds in kininogen to release Lys-bradykinin. Others have completely different functions, like Klk1b3 and Klk1b4 which are part of the 7S NGF complex, and Klk1b22 which can cleave β-NGF, drastically reducing its binding potential to its receptor. Members of this family with reduced or additional known activities are described in **Table 1**.

**Table 1:** Members of the mouse Kallikrein 1-related proteases with reduced or additional known activities.

Gene	Protein	Known functions
Klk1b3	γ-NGF	Cleaves a dipeptide from the β-NGF C-terminus binding with it and forming a part of the 7S NGF complex
Klk1b4	α-NGF	Inactive as a peptidase, but a stabilizing molecule of the 7S NGF complex
Klk1b9	EGFbp3	Epidermal growth factor-binding protein type C
Klk1b16	γ-renin	Can cleave the Leu-|-Leu bond in a synthetic 14-peptide renin substrate to produce angiotensin-I, but is inactive on angiotensinogen. Hydrolyzes Bz-Arg-p-nitroanilide
Klk1b21	mGK-21	Displays trypsin-like substrate specificity and shows activity towards casein, gelatin, fibronectin and IGFBP3
Klk1b22	EGF-BP A	β-NGF-endopeptidase, Epidermal growth factor-binding protein type A
Klk1b26	PRECE-2	Prorenin-converting enzyme. Cleaves mouse REN-2 prorenin at a dibasic site to yield mature renin
Klk1b27	mGK-27	Has chymotrypsin-like cleavage specificity with activity towards casein, gelatin, IGFBP3 and fibronectin but not towards laminin or collagens I, IV. Does not hydrolyze kininogen

NGF was originally described as an essential neurotrophin for the differentiation of the nervous system during development, but it is now recognized as having actions not restricted to the nervous system but also in immune system responses (12). In mice, the most abundant source of NGF are the submandibular salivary glands, where NGF is found mainly as a high molecular weight form, the 7S NGF complex (13). This complex contains the active β-NGF subunit, as well as Klk1b3 (mGK3) and Klk1b4 (mGK4) as the α- and γ- subunits (14–16). β-NGF can interact with its high affinity receptor, TrkA, or a low affinity, p75 receptor (17) to exert its biological activity. These receptors are expressed in many lymphoid organs, and neurotrophins, including NGF, have multiple documented immunomodulatory roles affecting among others the proliferation, differentiation, activation and chemotaxis of mast cells, B-cells, T-cells, monocytes/macrophages and other immune system cells (18). Despite that, information on the function of NGF with respect to SS is still limited.

### Aims

The aim of this study was to elicit a deeper understanding of the mechanism which leads to the observed SS-like morbidity in ERdj5^-/-^ mice through the identification of differentially regulated proteins within the afflicted SG tissue. Given the well-established sexual dimorphism that is exhibited in the submandibular salivary glands of mice, which has been described histologically and recently at the transcriptome level, we also aimed to describe it for the first time at the proteomic level, identifying the proteins that are prevalent at different abundances between sexes.

## Materials and Methods

### Mouse Cohorts

**Figure 1:**
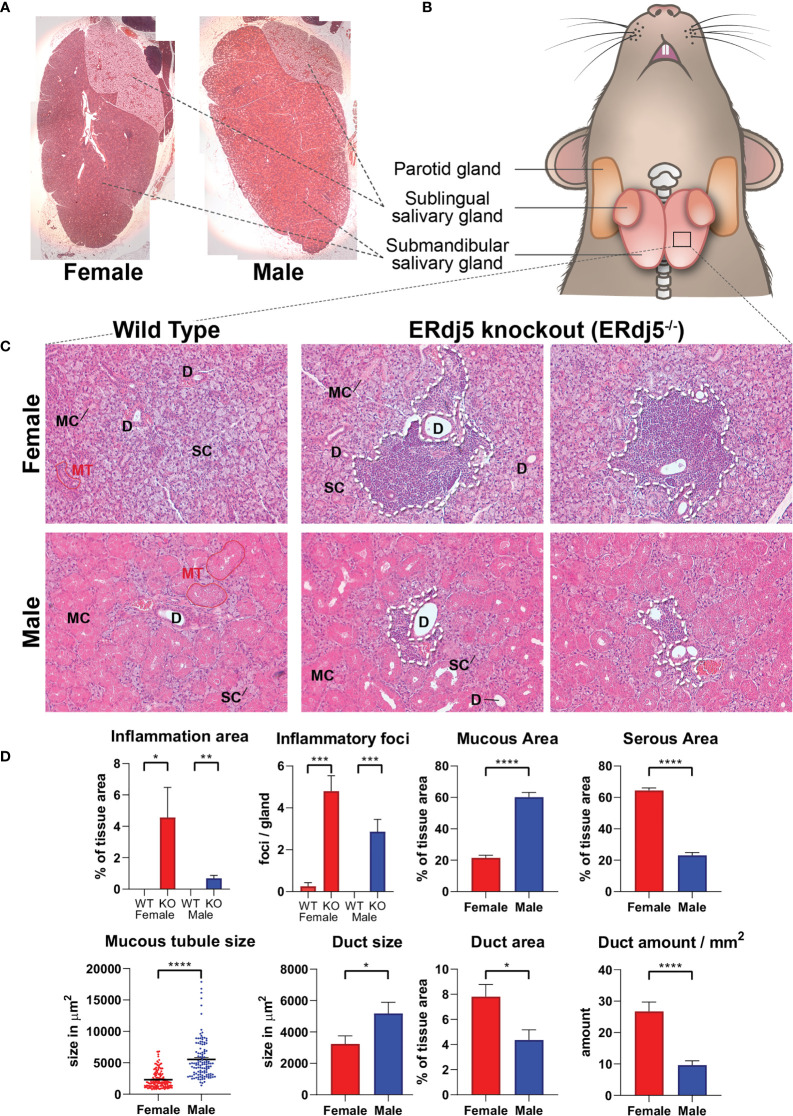
Histological image of mouse submandibular salivary glands and the inflammatory infiltrations in ERdj5 knockout mice. H+E stain. **(A)** Low magnification reconstruction of whole sublingual (top right side)/submandibular (main tissue) gland sections, illustrating the macroscopic differences between males and females. Original magnification: 25x. **(B)** Graphical representation of tissue samples. **(C)** Representative images for all study animals (n=6) under microscopic examination of submandibular gland features. Top Row: Female mice 7 months old. Bottom row: Male mice 7 months old. Inflammatory regions in KO mice are circumscribed with a dashed line. SC: serous cells, MC: mucous cells, D: ductal area, MT: mucous tubule. Original magnification: 200x. **(D)** Morphometric analysis. For inflammatory information, all tissue area of all animals was examined. For mucous/serous/ductal area quantification, 3 randomly selected microscopic frames were analysed and measured per study animal. For tubule size, 119 (from all male animals) and 153 (for female animals) tubules were measured. Data are presented as mean values ± SEM. Statistically significant differences according to t-test are indicated as *p < 0.05, **p < 0.01, ***p < 0.001 and ****p < 0.0001.

Submandibular salivary gland tissue samples (**Figure 1**) from twelve male and twelve female mice, aged 7-months, were used in this study, further divided according to their genotype in the following four cohorts: Female 129SV wildtype mice (n=6, cohort name FWT), female 129SV ERdj5 knockout mice (n=6, cohort name FKO), male 129SV wildtype mice (n=6, cohort name MWT) and male 129SV ERdj5 knockout mice (n=6, cohort name MKO). Tissue specimens from these animals had been previously histologically examined for the spontaneous development of inflammatory infiltration, and the 7-month timepoint was found to be when the lesions had been established in the submandibular SG tissues of all knockout animals. All animal experiments were conducted in full compliance to the Directive 2010/63/EU and approved by the Animal Care and Use Committee, Veterinarian Administration, Attiki prefecture (Protocol no. K/1279/11).

### Tissue Biopsy

The methods used for the acquisition of salivary gland tissue biopsies from the animals used in this study have been previously described (8). Briefly, mice were placed under ketamine/xylazine anaesthesia and operated upon for the excision of various tissue samples, including the submandibular salivary glands. The right submandibular SGs were snap frozen by immersion in dry-ice cold isopentane and stored at -80°C. These samples were used for mass spectrometry, qRT-PCR and western blotting. The left submandibular SGs were fixed in a 4% formaldehyde solution and subsequently processed with standard histology methods for embedding in paraffin blocks and are the samples used in this study for histochemical and immunohistochemical imaging.

### Histological Morphometry

Microscopy images of the Haematoxylin-Eosin stained submandibular gland sections of all the study animals were morphometrically analysed using the Image J software (FIJI distribution, Image J version 1.49v) as follows: 25x magnification photos were acquired for capturing and measuring the whole tissue area. These photos were used to reconstruct whole tissue images by collaging them in Adobe Photoshop software (version 21.0.2). 200x magnification photos were also acquired in 3 random fields per animal tissue section for the quantification of mucous cell area, serous cell area, mucous tubule size and duct amount and size. The same slides were scanned at 200x magnification and all fields containing inflammatory infiltrations were captured for the quantification of the amount of inflammatory foci and the total area of inflammation. Regions of interest (ROIs) were drawn in Image J defining the morphology of each measured modality in each image, which was subsequently measured for the determination of its area within the section, after calibrating with image size data for each magnification.

### FASP Preparation

For the preparation of salivary gland tissue samples for mass spectrometry analysis, we followed an adjusted method of Filtered Aided Sample Preparation (FASP) based on the Universal sample preparation method for proteome analysis (19). The detailed protocol is described in the **Supplementary Methods**.

### Liquid Chromatography Mass Spectrometry (LC/MS)

The lyophilized samples were reconstituted in 10μL 0.1% formic acid in ddH_2_O and analyzed through an LTQ Velos pro Orbitrap LC/MS instrument (Thermo Fisher, San Jose, CA) for the identification of peptides and their relative abundances. All tandem mass spectrometry (MS/MS) samples were analyzed using Sequest (Thermo Fisher; version 1.4.0.288). Scaffold (version 4.5.3, Proteome Software Inc., Portland, OR) was used to validate MS/MS based peptide and protein identifications. Additional information on the procedures of mass spectrometry is provided in the **Supplementary Methods**.

### qRT-PCR

Total RNA was isolated from the submandibular salivary glands of the same animals whose tissue samples were used for the proteomic analysis (n=6), and first strand cDNA was synthesized from the total RNA. qRT-PCR was performed in duplicates. The gene expression levels were normalized to the housekeeping gene PPIA (20) and calculated using the 2^–ΔΔCt^ method. Detailed protocols and the primer sequences used are listed in the **Supplementary Methods**.

### IHC Visualization

In order to determine whether the proteins that arose as the primary focus of our study (klk1b22 and NGF) were acting within the structures of the salivary gland tissue, in the areas of inflammation, or in other more specific sites within these areas, they were visualized immunohistochemically. Paraffin sections of the left submandibular gland from all mice in all groups (n=6) were stained with primary antibodies against mouse anti-klk1b22 and mouse anti-β-NGF and HRP-conjugated secondary antibodies. The protocol used for immunohistochemistry in this study and the antibodies are described in detail in the **Supplementary Methods**.

### Western Blot

Western blot of protein extracts from all the examined tissues (n=6) was performed in order to validate the difference in abundances between groups of the two main proteins of interest in this study, but also to ensure the protein specificity of the IHC images. Samples containing 30μg of total protein extract were loaded in a bis-acrylamide gel, transferred in a PVDF membrane and blotted against mouse anti-klk1b22 and mouse anti-β-NGF antibodies. The protocol and the antibodies used are detailed in the **Supplementary Methods** section.

### Statistical Analysis

Descriptive statistics on the salivary gland morphometric data were calculated using the GraphPad Prism software v.8.3.0. Group comparisons were also done within GraphPad Prism with unpaired parametric t-tests. The relative abundances of the identified proteins in LC/MS were estimated using both the exponentially modified protein abundance index (emPAI) and the normalized spectral abundance factor (NSAF) in Scaffold software v4.5.3 (see **Supplementary Methods**). Afterwards, 3 instances of t-tests were performed within Scaffold between group pairs, using the total spectra count, the NSAF calculated relative abundances and the emPAI calculated relative abundances. The group pairs for which protein abundance comparisons were performed where the female wildtype mice *vs* the male wildtype mice (FWT *vs* MWT), the female wildtype mice *vs* the female ERdj5^-/-^ mice (FWT *vs* FKO) and the male wildtype mice *vs* the male ERdj5^-/-^ mice (MWT *vs* MKO). A p value <0.05 in t-tests between groups in either emPAI or NSAF relative abundances was accepted as statistically significant. Additionally, proteins that were found to differ significantly between wild type animals and knockouts in both sexes were compared using a 2-way ANOVA analysis in GraphPad Prism. Group comparisons for the qRT-PCR results were analyzed using unpaired parametric t-tests in GraphPad Prism.

### Pathway Analysis

The resulting lists of proteins with significant differences in abundances between groups in these comparisons were further examined, for the determination of candidate targets for the development of a mechanistic model that could underly the observed phenotypic differences. The lists of these proteins were analyzed with the online functional protein association network tool STRING v11.0 (21) at www.string-db.org, as an aid for the identification of pathways and protein relations. In the case of female mice, the list of significantly changed proteins that we analyzed was enriched with β-NGF, which was found in our western blot and immunohistochemical experiments to be indeed downregulated in knockout animals, although its abundance was too small for significant differences to be detected in the mass spectrometry experiments. p values for significantly affected KEGG and reactome pathways were calculated automatically by the STRING tool.

## Results

### Histology – Sexual Dimorphism

The histological image of the examined tissues showed numerous inflammatory infiltrations in the knockout animals, which were more prominent and extensive in female knockout animals (**Figures 1C, D**). Furthermore, both wildtype and knockout animals presented a sexually dependent dimorphism regarding the morphology, size and distribution of acini, and the striated ducts. Specifically, males presented a histological image of more numerous and larger mucous tubules, also containing larger cells with more eosinophilic stain. Overall, the male submandibular gland tissue had a significantly higher content in mucous cells. In contrast, in the female tissues the serous acinar cells were significantly more prominent, comprising the most part of the observed tissue, and ducts were smaller, but also significantly more numerous, thus occupying a significantly larger percentage of the tissue area, although individually smaller (**Figure 1D**).

### Proteomic Data

**Figure 2:**
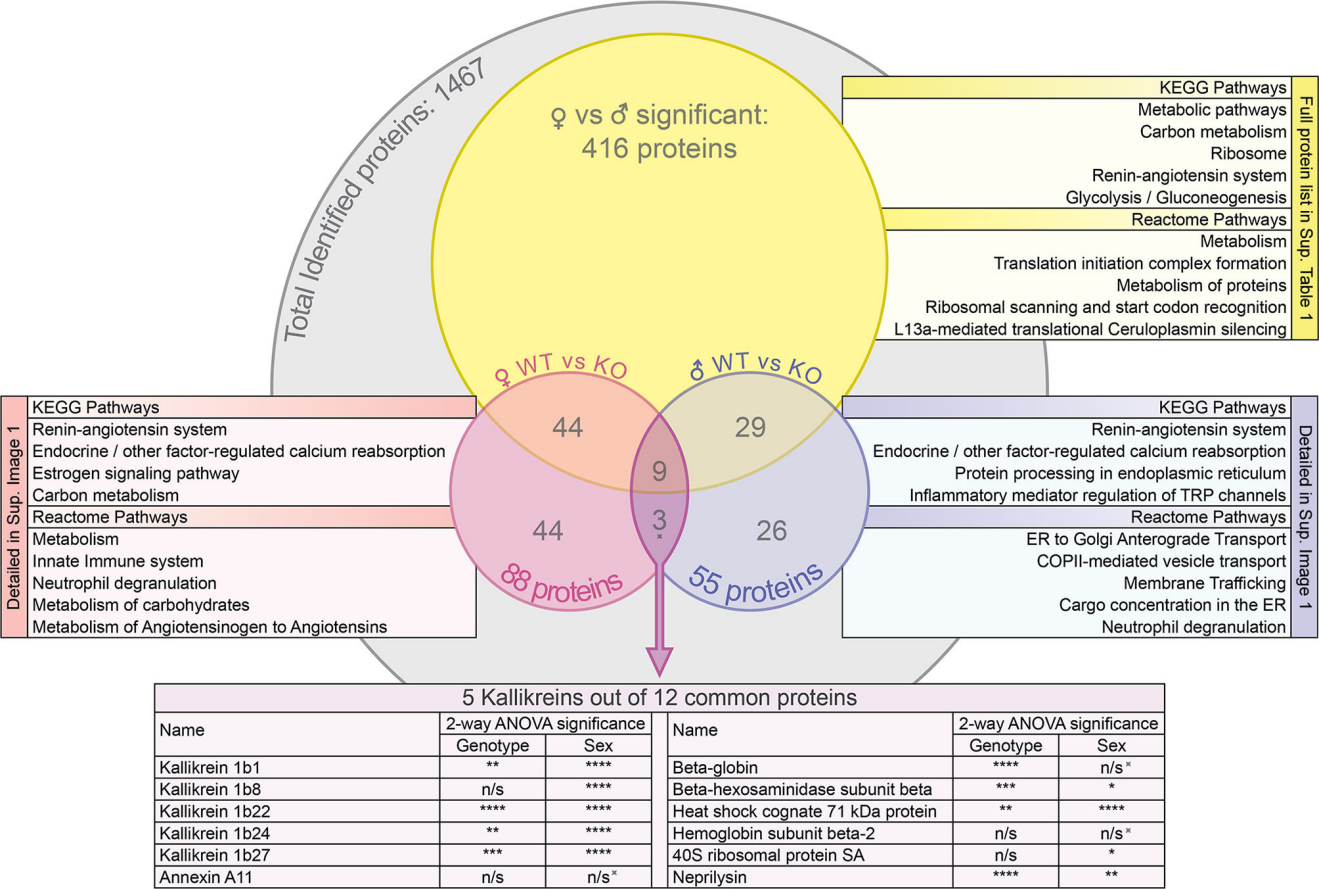
Venn diagram of the protein groups identified by the mass spectrometry proteomic analysis and the subsequent group comparisons. For the groups of proteins that were significantly different between FWT *vs* MWT, FWT *vs* FKO and MWT *vs* MKO animals (t-test p<0.05), the most significant KEGG pathways and reactome pathways for network enrichment in STRING interaction network analysis are listed. Additionally, 2-way ANOVA significance results of the NSAF quantification are presented for the commonly differing proteins (as initially identified by t-test) in both male and female WT *vs* KO: The spiked symbol indicates the 3 out of the 12 proteins that were not significantly different between sexes. n/s for not significant (p>0.05), *p < 0.05, **p < 0.01, ***p < 0.001 and ****p < 0.0001.

**Figure 3:**
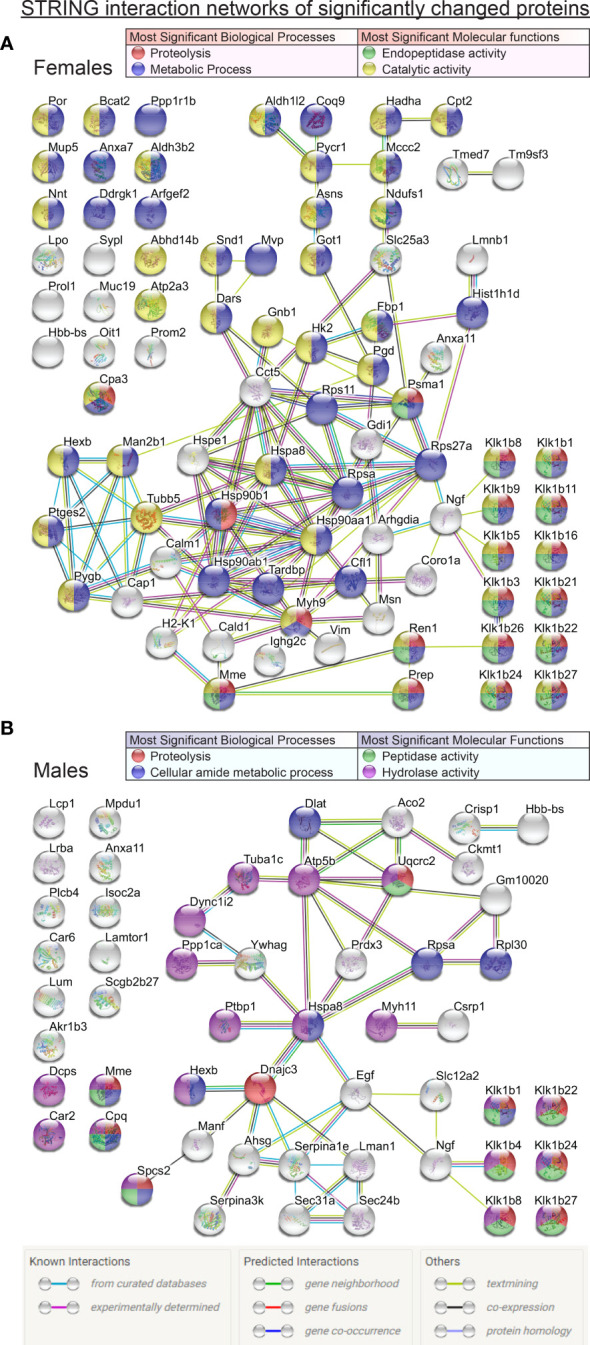
Comprehensive STRING interaction networks for the proteins that were found in significantly different relative abundances (t-test p<0.05) in the mass spectrometry proteomic data comparisons between FWT *vs* FKO animals **(A)** and MWT *vs* MKO animals **(B)**. Protein nodes are color coded according to their classification based on the most significant biological processes and molecular functions of the network proteins (legend tables). Edge color code in image legend.

**Figure 4:**
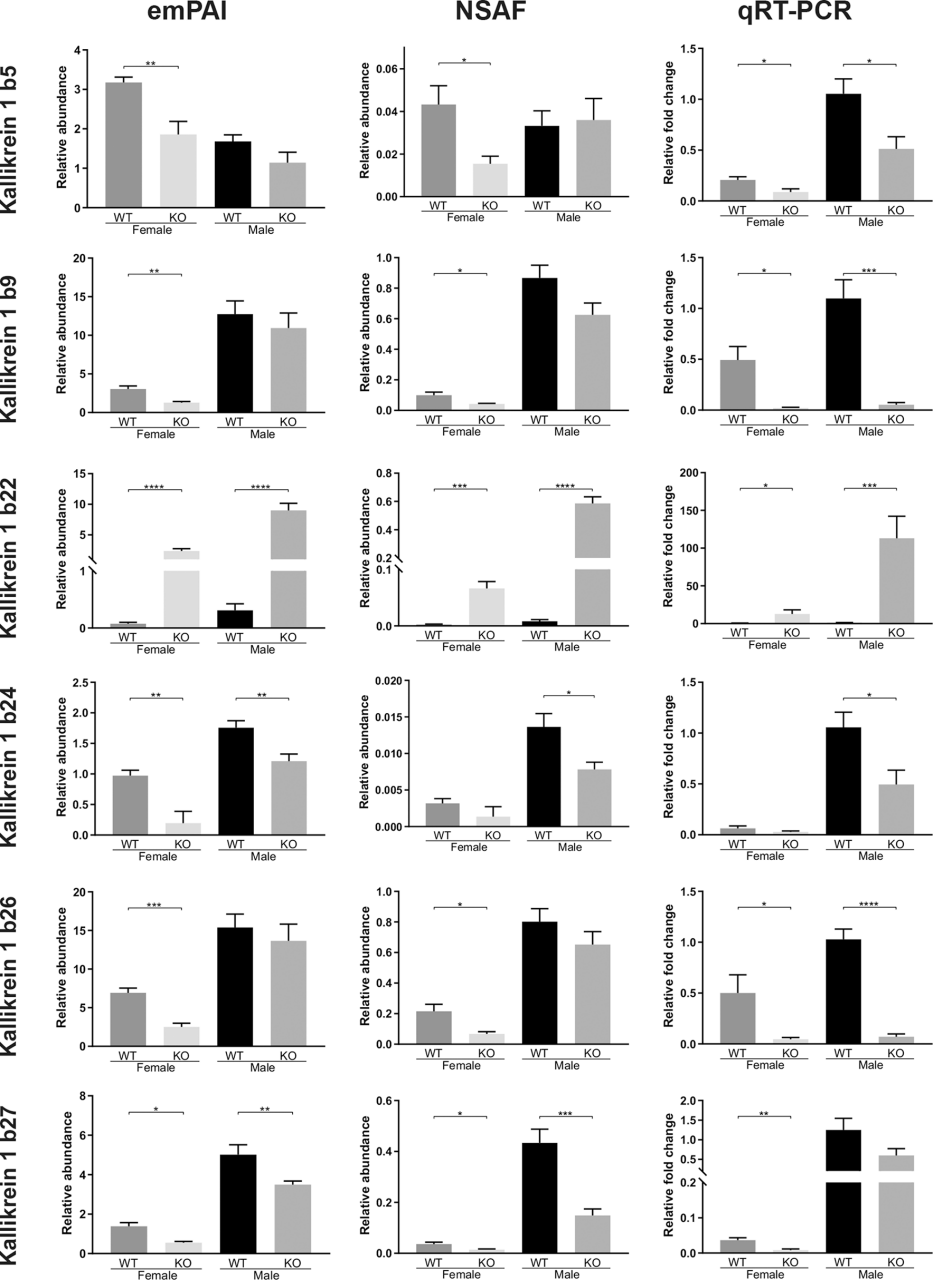
Quantitative comparisons of various Kallikrein 1-related proteases in the murine submandibular salivary gland tissue, at the protein level (NSAF and emPAI mass spec quantifications of relative abundances) and at the transcription level (qRT-PCR comparative fold change amounts). All animals in the proteomic analysis (n = 6) were also subsequently analyzed with qRT-PCR. Data are presented as mean values ± SEM. Statistically significant differences according to t-test between FWT *vs* FKO or MWT *vs* MKO groups are indicated as *p < 0.05, **p < 0.01, ***p < 0.001 and ****p < 0.0001.

Overall, 1467 proteins were identified in the mouse SG samples. Out of those, 1341 proteins were identified in the SGs of wildtype animals and 1396 in those of knockout animals. Exhaustive lists of the identified proteins and the comparison data of relative abundances between groups are provided in the supplementary spreadsheets (**Data Sheet 1**: FWT *vs* FKO, **Data Sheet 2**: MWT *vs* MKO, **Data Sheet 3**: FWT *vs* MWT). A result summary of the proteomic data group comparisons is visualized in **Figures 2**, **3** and **Supplementary Image 1**. In the comparison between FWT and FKO animals, 88 proteins were found to have a significant difference in relative abundances according to our established criteria (Statistical Analysis section). **Table 2** is a detailed list of these identified proteins along with the computed group difference t-test p-value. When comparing the relative abundances between MWT and MKO animals, 55 proteins were found to have a significant difference The detailed list of these proteins is illustrated in **Table 3**. Interestingly, an abundance of proteins that had a significant difference emerged when comparing wildtype male versus female animals, highlighting the already histologically observed sexual dimorphism: 416 proteins were found to have a significant difference (**Supplementary Table 1**). More than 28% of all identified proteins had a vast variability in their relative abundances between sexes, thus a direct comparison between wildtype and knockout animals disregarding the sex was impractical. Instead, we searched for proteins that were found differing in WT *vs* KO t-tests for both male and female group comparisons. We found that 12 proteins had significantly different abundances between WT and KO in both sexes. We additionally performed 2-way ANOVA analysis in the resulting list of common proteins with genotype (WT *vs* KO) and sex (male *vs* female) as the two variables. Eight of those twelve were indeed strongly validated in the stricter 2-way ANOVA analysis as genotype dependent; for those proteins, ANNOVA revealed a significant difference between WT and KO groups in both sexes (**Figure 2**). Interestingly, this short list of proteins was populated by multiple Kallikrein-1 peptidase family members: 5 out of the 12 proteins according to t-tests, 4 out of the 8 according to 2-way ANOVA were Klk1s. Out of those, only Klk1b22 was upregulated (**Figure 4** and **Supplementary Image 2**) while all the other Kallikreins (Klk1b1, Klk1b8, Klk1b24 and Klk1b27) were downregulated by the ERdj5 deletion. Notably, Klk1b22 had the highest confidence and the lowest p-value in both sexes between all the identified proteins (p<0.0001 in all metrics in males, p<0.00025 in all metrics in females) and was upregulated by the highest fold-change ratio (>29 fold in males, >26 fold in females). Four additional kallikrein family members have been excluded from this list, although they had significantly different spectra counts in both sexes, but the NSAF and emPAI quantification methods gave confidence values above our threshold for one sex. These were the Klk1b3, Klk1b9 and Klk1b21 proteases which were downregulated in female knockout animals, and Klk1b4 that was downregulated in male knockout animals. Finally, four Kallikrein proteases, namely Klk1b5, Klk1b11, Klk1b16 and Klk1b26 were found with significantly lower abundances in female KO mice while no significant differences were detected in male mice.

**Table 2:** Identified proteins with significant differences in computed relative abundances between female wildtype and female knockout animals (FWT *vs* FKO).

Name	NSAF p-value	emPAI p-value	KO/WT ratio NSAF | emPAI
Fructose-1,6-bisphosphatase 1	0.00014	<0.0001	0 | 0
NAD(P) transhydrogenase	0.00018	0.0017	0 | 0
Kallikrein 1-related peptidase b22	0.00025	<0.0001	26 | 31
Ig alpha chain C region	0.00031	0.00069	0 | 0.04
Neprilysin	0.00044	0.00055	0 | 0
Kallikrein 1-related peptidase b8	0.0022	0.00082	0.3 | 0.3
10 kDa heat shock protein	0.0025	0.0033	4.3 | 5.8
Aldehyde dehydrogenase	0.0031	0.0033	0.5 | 0.6
Coronin-1A	0.004	0.024	22 | 19
Mitoch. 10-formyltetrahydrofolate dehydrogenase	0.0043	0.0059	0.5 | 0.5
Kallikrein 1-related peptidase b3	0.0071	0.02	0.4 | 0.3
Renin-2	0.0077	0.00032	0.2 | 0.2
Mast cell carboxypeptidase A	0.0083	0.02	0.3 | 0.2
Lysosomal alpha-mannosidase	0.01	0.0047	0.2 | 0.2
Hemoglobin subunit beta-2	0.013	0.0083	0 | 0
Annexin A7	0.014	0.0085	INF | INF
Pyrroline-5-carboxylate reductase 1	0.017	0.01	0.5 | 0.5
Kallikrein 1-related peptidase b26	0.02	0.0004	0.3 | 0.4
Heat shock cognate 71 kDa protein	0.022	0.047	0.8 | 0.8
Protein Ighg2c (Fragment)	0.023	0.0082	INF | INF
Kallikrein 1-related peptidase b5	0.024	0.0035	0.4 | 0.6
Prolyl endopeptidase	0.025	0.013	0.4 | 0.3
Kallikrein 1-related peptidase b21	0.028	0.014	0 | 0.2
MCG10343, isoform CRA_b	0.028	0.0085	2.4 | 2.4
Kallikrein 1-related peptidase b9	0.03	0.0046	0.4 | 0.4
Hexokinase	0.03	0.039	INF | INF
NADH-ubiquinone oxidoreductase 75 kDa subunit	0.031	0.023	1.8 | 1.6
Synaptophysin-like protein 1, isof. 2	0.032	0.014	0.5 | 0.4
Alpha/beta hydrolase domain-containing protein 14B	0.032	0.0079	3.6 | 4.5
NADPH–cytochrome P450 reductase	0.032	0.031	INF | INF
Kallikrein 1-related peptidase b27	0.033	0.0038	0.4 | 0.4
Adenylyl cyclase-associated protein 1	0.033	0.013	1.7 | 1.8
Brefeldin A-inhibited guanine nucleotide-exchange protein 2	0.037	0.025	INF | INF
Ig mu chain C region	0.04	0.019	16 | 14
Trifunctional enzyme subunit alpha	0.044	0.035	1.7 | 1.6
Major vault protein	0.046	0.031	3.5 | 3.5
Glycogen phosphorylase, brain form	0.047	0.033	1.6 | 1.7
Staphylococcal nuclease domain-containing protein 1	0.0036	0.93	0.8 | 1
Beta-globin	0.0061	0.094	95 | 3.2
Histone H1.3	0.0064	0.15	1.5 | 1.4
Protein FAM3D	0.0099	0.05	0.4 | 0.4
Calmodulin	0.014	0.12	0.4 | 0.6
Lamin-B1	0.016	0.14	2.3 | 1.8
Major urinary protein 5	0.022	0.063	0 | 0
Branched-chain-amino-acid aminotransferase	0.023	0.085	0.5 | 0.6
Moesin	0.025	0.089	4.8 | 3
Tubulin beta-5 chain	0.028	0.063	2.7 | 1.9
Caldesmon 1	0.03	0.15	2.3 | 2.1
Calcium-transporting ATPase	0.031	0.37	0.8 | 0.9
Endoplasmin	0.034	0.65	0.6 | 0.9
Vimentin	0.035	0.086	2 | 2.1
Protein Lpo	0.036	0.12	0.6 | 0.7
Ubiquinone biosynthesis protein COQ9	0.036	0.15	5 | 3.3
Proteasome subunit alpha type-1	0.038	0.086	0.2 | 0.2
H-2 class I histocompatibility antigen, K-Q alpha chain	0.038	0.071	8.7 | 6.2
Carnitine O-palmitoyltransferase 2	0.045	0.058	4 | 2.9
Prostaglandin E synthase 2	0.045	0.089	INF | INF
Kallikrein 1-related peptidase b11	0.047	0.054	0.3 | 0.7
Kallikrein 1-related peptidase b16	0.047	0.095	0.5 | 0.4
T-complex protein 1 subunit epsilon	0.049	0.093	2.8 | 3.2
Prominin-2	0.05	0.0066	0.5 | 0.6
Transmembrane 9 superfamily memb. 3	0.052	0.04	0.1 | 0.09
Aspartate–tRNA ligase, cytoplasmic	0.053	0.013	2.7 | 3.9
Mitoch. methylcrotonoyl-CoA carboxylase β chain	0.054	0.019	2.7 | 2.6
Ig gamma-2A chain C region, A allele	0.057	0.043	0 | 0
DDRGK domain-containing protein 1	0.058	0.041	0.4 | 0.4
Beta-hexosaminidase subunit beta	0.069	0.044	0.3 | 0.3
Protein Prol1	0.071	0.015	0.3 | 0.3
Kallikrein 1-related peptidase b1	0.075	0.0079	0.5 | 0.4
Submandibular gland protein C, isof. 3	0.075	0.012	2.1 | 2.3
40S ribosomal protein SA	0.077	0.046	0.6 | 0.6
Protein phosphatase 1 regulatory subunit 1B	0.078	0.02	1.5 | 1.6
MCG16669, isoform CRA_f	0.079	0.049	2.7 | 3.3
40S ribosomal protein S11	0.1	0.041	0.4 | 0.5
Asparagine synthetase	0.11	0.023	0.5 | 0.5
Protein Tmed7	0.11	0.037	0.4 | 0.3
Ubiquitin-40S ribosomal protein S27a	0.12	0.032	1.6 | 1.5
Myosin-9	0.13	0.027	1.2 | 1.4
Heat shock protein HSP 90-beta	0.14	0.02	1.3 | 1.5
Rab GDP dissociation inhibitor alpha	0.17	0.048	0.7 | 0.6
Rho GDP-dissociation inhibitor 1	0.23	0.038	1.5 | 1.5
Kallikrein 1-related peptidase b24	0.24	0.0039	0.4 | 0.2
Guanine nucleotide-binding protein G(I)/G(S)/G(T) subunit beta-1	0.24	0.027	0.5 | 0.4
Aspartate aminotransferase, cytoplasm.	0.24	0.037	1.4 | 1.6
6-phosphogluconate dehydrogenase	0.24	0.045	0.7 | 0.6
Annexin A11	0.27	0.012	1.4 | 2
Heat shock protein HSP 90-alpha	0.32	0.027	1.3 | 1.9
Cofilin-1	0.51	0.015	1.2 | 1.8

p values < 0.05 were considered significant. INF, infinite ratio (denominator = 0).

**Table 3:** Identified proteins with significant differences in computed relative abundances between male wildtype and male knockout animals (MWT *vs* MKO).

Name	NSAF p-value	emPAI p-value	KO/WT ratio NSAF | emPAI
Kallikrein 1-related peptidase b22	<0.0001	<0.0001	71 | 29
Carbonic anhydrase 6	0.00016	0.016	3.4 | 1.5
Neprilysin	0.00022	0.0026	0.07 | 0.07
Kallikrein 1-related peptidase b27	0.00076	0.018	0.3 | 0.7
Myosin-11	0.00093	0.0039	1.5 | 1.2
ABPBG27	0.0012	0.019	3.8 | 2.7
Mannose-P-dolichol utilization defect 1	0.0016	0.0011	5.2 | 4.9
Beta-globin	0.0021	<0.0001	INF | 24
Carbonic anhydrase 2	0.0033	0.0013	0.2 | 0.2
Alpha-2-HS-glycoprotein	0.0035	0.0071	2 | 1.7
Beta-hexosaminidase subunit beta	0.0041	0.0015	0.3 | 0.3
m7GpppX diphosphatase	0.009	0.044	3 | 2.3
Lumican	0.009	0.0051	0.6 | 0.5
Ragulator complex protein LAMTOR1	0.011	0.011	0.2 | 0.2
Dihydrolipoyl transacetylase	0.012	0.021	2.8 | 2.5
60S ribosomal protein L30	0.013	0.044	7.3 | 5.4
Cysteine-rich secretory protein 1	0.013	0.013	0 | 0
Carboxypeptidase Q, Isof. 2	0.013	0.031	0.2 | 0.3
LRBA	0.015	0.014	0.2 | 0.2
Pro-epidermal growth factor	0.016	0.0086	0.6 | 0.6
TRX-dependent peroxide reductase	0.018	0.025	5.4 | 5.1
Kallikrein 1-related peptidase b24	0.018	0.0073	0.6 | 0.7
Tubulin alpha-1C chain	0.019	0.017	1.4 | 1.4
40S ribosomal protein SA	0.02	0.039	2.4 | 2.1
DnaJ homolog subfamily C member 3	0.022	0.011	0.3 | 0.2
Aldose reductase	0.023	0.033	4.7 | 4.6
Protein Sec24b	0.023	0.033	INF | INF
Alpha-1-antitrypsin 1-5	0.041	0.049	7.2 | 4.8
Polypyrimidine tract-binding protein 1	0.042	0.03	2.4 | 2.3
Aconitate hydratase, mitochondrial	0.00094	0.12	1.4 | 1.1
ATP synthase subunit beta	0.0025	0.24	1.3 | 1.2
Hemoglobin subunit beta-1	0.003	0.89	1.7 | 1
Solute carrier family 12 member 2	0.0045	0.05	2.3 | 1.8
Kallikrein 1-related peptidase-like b4	0.0048	0.26	0.6 | 0.8
Kallikrein 1-related peptidase b8	0.0077	0.46	0.7 | 0.8
Kallikrein 1-related peptidase b1	0.011	0.47	0.6 | 0.9
PPP1CA	0.022	0.12	2.6 | 1.9
Beta-nerve growth factor	0.022	0.28	0.3 | 0.7
Isochorismatase domain containing 2a	0.025	0.09	3.6 | 2.6
Phospholipase C beta 4	0.025	0.085	1.9 | 1.5
Annexin A11	0.026	0.058	2.5 | 2.2
Protein ERGIC-53	0.028	0.073	5.1 | 4.4
14-3-3 protein gamma	0.039	0.22	1.8 | 1.3
Ribosomal protein L15	0.039	0.082	3.2 | 2.8
Plastin-2	0.041	0.12	2.5 | 2
Signal peptidase complex subunit 2	0.045	0.092	3.3 | 3.2
Cytochrome b-c1 complex subunit 2	0.047	0.095	1.8 | 1.6
Mesencephalic astrocyte-derived NF	0.048	0.1	1.8 | 1.7
Serine protease inhibitor A3K	0.072	0.036	0.5 | 0.4
Creatine kinase U-type, mitochondrial	0.078	0.024	0.7 | 0.5
Protein transport protein Sec31A, Isof.2	0.094	0.035	0.5 | 0.4
Heat shock cognate 71 kDa protein	0.18	0.0023	0.8 | 0.6
Cysteine and glycine-rich protein 1	0.48	0.023	0.8 | 0.6
Cytoplasmic dynein 1 interm. chain 2	0.57	0.033	0.9 | 0.6
Hemoglobin subunit beta-2	0.9	0.0019	1 | 0.2

p values < 0.05 were considered significant. INF, infinite ratio (denominator = 0).

A Kallikrein interacting molecule and substrate for Kallikrein protease activity is the Nerve Growth Factor (NGF), and more specifically the mouse salivary 7S NGF complex. In our proteomic analysis data, the β-NGF subunit was also significantly downregulated in MKO mice compared to MWT mice according to the NSAF quantification method (**Table 3**, NSAF p=0.022, 3.3-fold decrease). However, β-NGF in wildtype animals was found at a 20-fold lower abundance in female mice according to NSAF, or at a more moderately, but still significantly decreased abundance according to emPAI (**Supplementary Table 1**, NSAF p=0.0029, emPAI p=0.0057). This significant difference was probably the reason why β-NGF was detectable at very low levels in FWT mice, and nearly undetectable in FKO mice, thus not producing results of statistical significance in females (**Supplementary Sheet 1**).

### Protein Interaction Network

The analysis of the lists of proteins with significantly different abundances between wildtype and knockout animals with the STRING online analysis tool revealed significant pathways, reactomes and molecular functions of interest. **Figure 2** lists the most significant KEGG pathways and reactome pathways that the proteins of interest were found to be part of. The renin-angiotensin system arose as the most significant in both sexes, followed by the endocrine/other factor-regulated calcium reabsorption pathway. Other pathways of significant interest in both sexes included metabolic pathways, inflammatory mediation pathways, the innate immunity system and neutrophil degranulation pathways. Expectedly, ER trafficking and processing pathways were also important but rose on top of the most significant pathway list only in males. The comprehensive networks of all the proteins of interest are graphically illustrated in **Figure 3**. In both sexes, the most abundant groupings of proteins based on molecular function and biological processes were those with peptidase or hydrolase activity, naturally participating in metabolic and proteolytic functions. β-NGF stands out as a possible connection linking the differentially expressed kallikreins to the rest of the network. At the center of the network in both sexes reside proteins of the Heat shock family, and specifically Heat shock cognate 71 kDa protein (Hspa8) that was significant in both male and female group comparisons, and was linked to β-NGF through 1 intermediate vertex, EGF in males and Ubiquitin-40S ribosomal protein S27a (Rps27a) in females. An alternative connection to β-NGF in females included the intermediary vertex of 40S ribosomal protein SA (Rpsa), which interestingly was also a protein found significantly changed in both males and females, and found to be significantly impacted by sex in our ANOVA analysis. In male mice though, a possible unidentified link prevented it from being connected to the β-NGF node. Regarding other proteins that were significantly changed in both group comparisons, Neprilysin (Mme) was connected through Renin (Ren1) to the kallikrein group in females, but was not part of any network in males. Annexin A11 (Anxa11) did not form part of any network in males, and in females it was a poorly connected node that did not seem to form relationships of significance to the network. Beta globin (Hbb-bs) was not a part of any significant network in any sex. Lastly, Beta-hexosaminidase subunit beta (Hexb) was part of a completely separate network in females, and a blind node in males. Interestingly though, in males, its connection to the network was *via* DnaJC3 to the central Hspa8. DnaJC3 is a protein belonging to the J-domain family of proteins, as is ERdj5 (DnaJC10).

### RT-PCR Quantification of Transcription Levels

After the proteomic analysis of the SG samples, we selected the proteins of highest confidence and interest that differed between groups and proceeded to validate the results with independent methods that would also allow us to determine whether the differences in abundances were due to pre- or post-translational processes. We thus explored the expression profile of selected kallikrein proteases with qRT-PCR. Overall, the results were in agreement to the proteomic results for most of the tested kallikreins apart from some exceptions. Importantly, the differences that had the highest confidence in our proteomic analysis, were indeed confirmed with a high statistical significance at the transcription level: Klk1b22 was again, the only kallikrein found to be upregulated in the KO mice in both sexes, with a bigger than 20-fold change ratio (FWT *vs* FKO: p=0.04, 22-fold increase. MWT *vs* MKO: p=0.0033, 99.1-fold increase). The other kallikrein family members with significant results, largely consistent with the proteomic data are Klk1b5, Klk1b9, Klk1b24, Klk1b26 and Klk1b27 (**Figure 4**). Klk1b5 was downregulated in females (2.35-fold, p=0.0272) like it was at the proteome level, but also in males (2-fold, p=0.0162). The same result was found for Klk1b9, validating the downregulation in females (24.6-fold, p=0.0102) but we also measured a significant difference in males (20.6-fold, p=0.0002). Likewise, the Klk1b26 data validated the downregulation in FKO animals (10.9-fold, p=0.0482) but also showed a downregulation in MKO mice (14.7-fold, p<0.0001). The Klk1b24 data confirmed the downregulation in male mice (2.1-fold, p=0.0209) but did not validate the difference that was detected between female mice in the NSAF protein abundance. Inversely, the Klk1b27 PCR data confirmed the downregulation in FKO mice (4.6-fold, p=0.0064) but did not confirm the downregulation that was found in the MKO by both the NSAF and emPAI abundance quantifications. The results of the rest of the kallikreins that were tested (Klk1b1, Klk1b3, Klk1b4, Klk1b8, Klk1b11, Klk1b16, Klk1b21) are presented in the **Supplementary Image 2**. Of those, only Klk1b8 failed to validate at the transcription level the highly significant downregulation that was detected in the proteome of FKO mice, but did nonetheless have transcription levels that validated its downregulation in male mice (2.2-fold, p=0.0079).

### IHC Visualization of Klk1b22 and β-NGF

**Figure 5:**
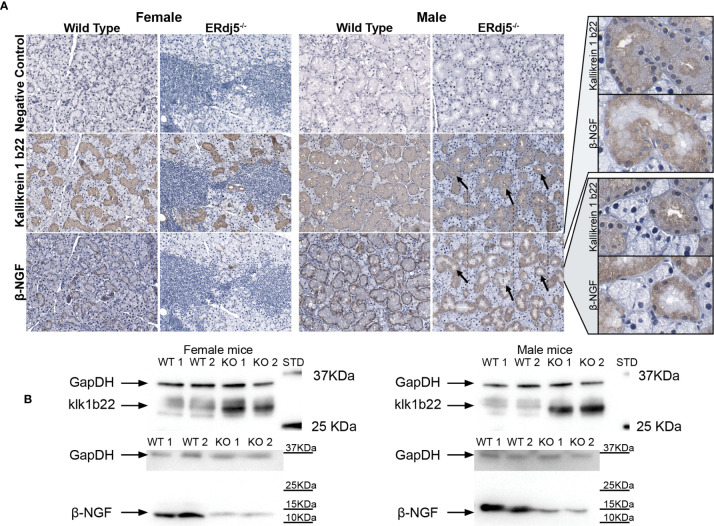
Detection of Kallikrein 1b22 and β-NGF in the murine submandibular salivary gland tissue with antibodies. **(A)** Representative images (n = 6) of *in situ* immunohistochemical visualization of kallikrein 1b22 and β-NGF in co-localized paraffin sections of murine salivary glands. All mice within groups presented the same respective staining pattern. Negative control sections were treated with Ab diluent without primary Ab, followed by the secondary Ab incubation, DAB and Hematoxylin staining. Positive signal: Brown, counterstain: Blue, hematoxylin. The black arrows indicate the same tissue regions in different stains, where the positive signal for kallikrein 1b22 coincides with a lack of positive stain for β-NGF. Rightmost column: Increased magnification of these regions for both Kallikrein 1b22 and β-NGF. **(B)** Representative western blot images (n = 6) for the detection of Kallikrein 1b22 (approximately at 28kD) and β-NGF (approximately at 12kD). Samples for female and male mice were run at individual gels in the case of kallikrein 1b22, and in the same gel but with different exposure times for each sex in the case of β-NGF.

Staining salivary glands with antibodies against Klk1b22 and the β subunit of the 7S NGF complex, we visualized the localization of these two proteins within the submandibular SGs of all study animals (n=6) (**Figure 5A**). Notably, both proteins were localized mainly in the mucous cells and not at all in the serous cells. Additionally, Klk1b22 was localized in the ductal cells, but that was not the case for β-NGF whose staining was exclusive to the mucosa. The inflammatory lesion regions had no positive signal, neither for Klk1b22 nor for β-NGF. In male KO mice, Klk1b22 within the mucous cells localization presented a polarization pattern: The regions of high Klk1b22 signal were in the basal side, oriented towards the ductal lumen and away from the cell nucleus. Such a pattern was not obvious in the WT male animals. Also, this pattern was not noticed in the ductal cells of female mice samples in which the Klk1b22 signal appeared both stronger and uniform. Additionally, in both male and female mice respectively, KO animals had a stronger Klk1b22 signal compared to WT. Although not quantifiable through immunohistochemical imaging, the difference in Klk1b22 abundance between male and female mice could at least in part be attributed to the histological differences between the two sexes, with the submandibular salivary glands of female mice having notably less mucous cells, which were the sources of positive signal, per examined area, but also smaller ducts in general.

Regarding the staining against the β-NGF subunit in males, the source of positive signal was the mucous cells that were positive for Klk1b22. Interestingly, β-NGF staining also presented a cellular polarization pattern in its localization, but opposite of that of Klk1b22; β-NGF was detected on the apical, nuclear side of the cell, juxtaposed to the basal surface. Moreover, in closely colocalized sections it was apparent that cellular regions with high Klk1b22 signal were negative for β-NGF staining. Also, in MWT animals the β-NGF signal localization was tighter and stronger towards the periphery of the duct, while in MKO animals the staining was fainter and more diffuse. In female wildtype animals the localization pattern was like their male counterparts, with the difference of the relative scarcity and smaller size of the mucous cells due to the observed histological sexual dimorphism. Moreover, staining appeared to be less intense, although it retained the tight localization towards the nuclear-side cellular membrane, distant from the lumen. In female ERdj5^-/-^ animals on the other hand, the β-NGF signal was minimal, restricted to the periphery of some ducts and only in a faint manner if any.

### Western Blot Validation

We also performed western blot in order to ensure that there was no nonspecific positive signal that could be interfering in the IHC images, and also as an additional validation of the differences observed in the proteomic analysis and the transcriptional quantification. Indeed, the results of the western blot for Klk1b22 validated its high upregulation in both male and female KO animals, in contrast to WT of both sexes (**Figure 5B**). Additionally, the difference in Klk1b22 abundance between FKO and MKO mice was observed by the longer exposure time that was needed in order to visualize the bands in female animals (double exposure time). The inverse pattern was observed for β-NGF in the same animals, where WT animals of both sexes had substantially higher amounts of β-NGF compared to the KO animals. Western blot being a substantially more sensitive method, the difference in β-NGF abundance between FWT and FKO animals was evident in the western blots, although it was only hinted at by the IHC images, and not detectable in the proteomic analysis due to the low β-NGF abundance in female mice.

## Discussion

**Figure 6:**
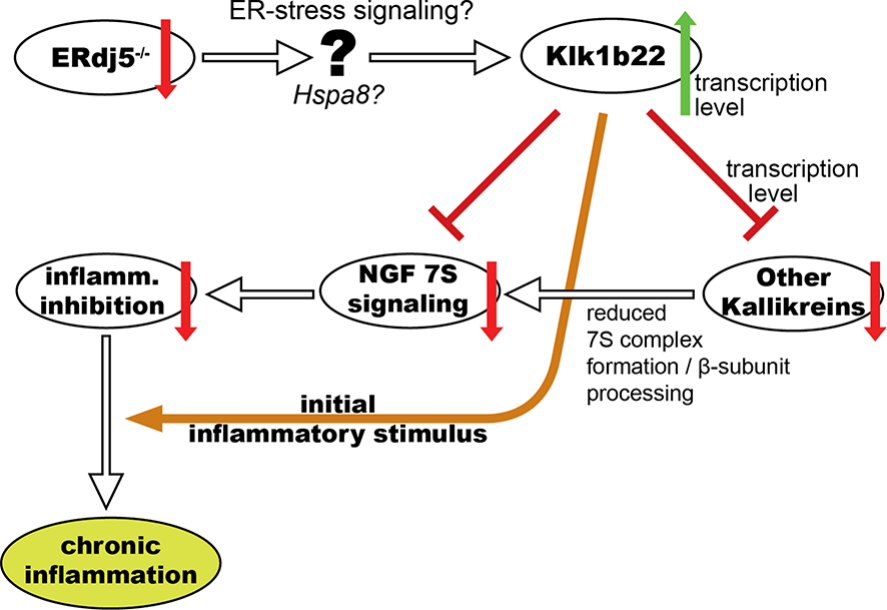
Schematic representation of our working mechanistic model for the involvement of ERdj5 ablation, ER-stress and kallikrein deregulation in the development of an SS-like phenotype in mice through NGF.

In this study we have explored the proteomic profile of the submandibular salivary glands of ERdj5 knockout and wildtype mice of both sexes in order to investigate the molecular basis of the observed SS-like pathology in the ERdj5^-/-^ mouse model. After identifying proteins that are potentially involved in the morbid phenotype, we proceeded to validate those results with independent methods which also provided evidence on the nature of the regulation and the cellular localization of the target molecules. Importantly, since the immunohistochemical detection of the two main proteins of interest in this study was limited to the mucosal and ductal areas of the tissue and not within the inflammatory lesions, the observed differences cannot be attributed to the established different content of immune cells between the groups. These analyses have allowed us to form a working mechanistic model which connects ER-stress to observable differences in the expression of specific proteins that can explain the autoimmune pathology development (**Figure 6**).

At the same time, this study provides a comprehensive proteomic report for the well-established sexual dimorphism of the murine salivary glands (22). To our knowledge, this is the first study documenting this at the proteome level. Two previous studies have focused on the sexual dimorphism in murine SGs at the transcriptome level, one using microarray analysis (23), and one using RNA deep sequencing (24). The results from the latter study are in notable agreement with our findings, especially regarding the key proteins of interest in our experiments. Specifically, all kallikrein 1b family members that were detected as differing in our study and validated by qRT-PCR were also prominently different in the RNA-seq transcriptome study (24). Additionally, the differential expression of NGF between males and females was a common finding, as was the beta-hexosaminidase subunit beta and two members of the Serpin protease inhibitor family. In the results of the RNA microarray study, there was important agreement with our data on the differential expression of EGF, NGF (higher in males) and Sialyltransferase 4 (higher in females). Overall, it is important that NGF, which arose as a key molecule in our study is also confirmed by both relevant studies in the literature, and that EGF was also confirmed in at least one study. Since these molecules, including the kallikrein protease family, were found to be important factors in the difference between our knockout, SS-phenotype mice and wildtype animals, their differential baseline levels between sexes is a highly plausible explanatory basis for the sexual discrepancy in the prevalence and severity of inflammatory lesions in the SGs of our mouse model, and by extension a possible explanation for the high female bias of Sjögren’s syndrome in the human population.

A similar study that explored the sexually dimorphic proteomic and transcriptomic profile in mouse secreted saliva had the kallikrein protease family prominently different between males and females (25). Notably though, although they detected all the kallikrein 1b members that arose as significant in our study also, they found them in significant higher abundances in females, while our results in the tissue samples have a high confidence of the exact opposite bias, ranging from 3.4 to 23-fold more abundant in males. Our data are in agreement with the secretory data only in the case of Kallikrein 1, which was also of 10-fold lower abundance in males. When focusing on the tissue rather than on the secreted saliva, it is noteworthy that a previous transcriptomic study is in agreement with our proteomic data regarding the sex bias direction (kallikrein 1b family RNA was higher in males) (24). Because as described below, at least some of these proteases have autoantigenic potential, the difference in their SG tissue *vs* saliva abundance suggests an interesting possibility, that the secretory status of these proteins could be the reason for the observed pathologies in females. It may be the case that male mice retain these proteases within the cellular bounds, while the females tend to secrete them, exposing them to the immune system which may have autoreactive responses towards them.

The Kallikrein 1b protease family that was significant in our study both as major contributor of sex dimorphism but also as key players in the knockout *vs* wildtype comparisons does not have one-to-one human analogs. This leaves open for investigation the prospect that other proteases, driven by the same ER-stress – NGF mechanisms might serve similar roles in the human pathology. Cathepsin S is such a potential, but not exclusive, target since it has been implicated in antigen presentation and autoimmune reactions including SS (26). A human salivary gland microarray mRNA analysis of SG sexual dimorphism provided some other candidates in the peptidase category, namely beta-secretase 2, signal peptidase complex, endoplasmic reticulum metallopeptidase 1, isoaspartyl peptidase/L-asparaginase, complement factor I, cytosolic carboxypeptidase 2, and STAM-binding protein (27). Although this list might not be exhaustive, due to the microarray nature of the study and the limitation of being able to sample only minor salivary glands in humans, it is still noteworthy that this study found seven proteins whose homologues in our mice were also sexually dimorphic (beta-hexosaminidase subunit beta, lysosomal alpha-mannosidase, pyrroline-5-carboxylate reductase 1, transmembrane 9 superfamily member 2, prohibitin, membrane-associated progesterone receptor component 2 and 4-trimethylaminobutyraldehyde dehydrogenase). Out of those seven, the first three were also found to significantly differ in our WT *vs* KO comparisons (**Figure 3**). Also, the closely related transmembrane 9 superfamily member 3 and various other aldehyde dehydrogenases were significantly different between female wildtype *vs* knockout animals. Overall, out of 7 homologue matches of the human sexual dimorphism proteome, 5 seem to be involved in the inflammatory phenotype of our mouse model. We believe that deeper investigation of those proteins has a great potential to provide significant insights on a complementary pathway to SS pathology to the one we have focused on in this study.

Previous studies have demonstrated that indeed, mouse glandular kallikreins can act as immunomodulatory molecules, enhancing the proliferation of specific lymphocytes while being cytotoxic to others (28). In our experimental data, kallikrein 1b22 stood out not only for the high confidence of the differences between wildtype and knockout animals of both sexes but also for the big fold induction it displayed. Moreover, it was the only member in the kallikrein family to be upregulated, when inversely, every other kallikrein was downregulated in the absence of ERdj5. Importantly, after it had been recognized that subcutaneous injections of salivary or lachrymal gland extracts were able to induce an inflammatory response and an SS-like phenotype in mice (29–31) and rats (32), kallikrein 1b22 has been isolated and identified as the sole extract fragment to be able to induce the SS-like phenotype in rats (33). Also, in the NOD Aec1/Aec2 mouse model for SS, a correlation of local IL-17 sequestration in the salivary gland and the reduction of tissue levels of Klk1b22 has been shown, further strengthening the connection between mechanisms relevant to SS and this particular molecule (34). Although not conclusive, this also implies that our results regarding the implication of kallikreins in the SS phenotype are not mouse strain specific, but may potentially apply to other models of the disease. For these reasons, we focused on this molecule in our subsequent analysis, looking at its potential downstream effects and how they could be leading to the observed immune responses. A plausible scenario is that this molecule can be acting as an autoantigen itself, being recognized by the immune system and eliciting its reaction. Although the mass spec method utilized in this study is not sensitive enough for the thorough analysis of the innate and adaptive inflammatory milieu and its dysregulation in SS, it is interesting that our previous research has demonstrated substantially higher IL-10 and IL-23 levels in aged KO female animals that manifest locally and of IL-18 in the male mice that were included in this study. Systemically, we have also found an age progressing IL-17A upregulation in male KO mice and an upregulation of IL-18 and IL-23 in KO female mice (8). We have additionally identified in the present study a small group of deregulated proteins that are implicated in innate or adaptive immune processes (**Supplementary Table 2**), although these findings cannot paint a complete picture without further research. Looking at the known functions of Klk1b22, another prospect also stands out, that of its ability to cleave the mature β-NGF (13) (remove 8 N-terminal a.a.) producing a form of β-NGF with > 50-fold less affinity to its TrKA receptor (35). Human NGF has been reported to be able to suppress inflammatory responses through this specific receptor (36). Thus, increased Klk1b22 activity could be driving the inactivation of NGF and its immunosuppressing function, thus enabling aberrant immune responses.

This hypothesis is bolstered by the fact that in the salivary glands of mice, the 7S NGF complex is an α2-β2-γ2 complex in which the β-NGF dimer (the active neurotrophin) is associated with two α-NGF and two γ-NGF subunits (14). The α and γ subunits belong to the glandular kallikrein family of serine proteinases. The α-NGF is an inactive kallikrein serine proteinase, Klk1b4 (mGK4) (16), whereas the γ-NGF subunit is an active serine proteinase, namely Klk1b3, capable of processing the precursor form of β-NGF by cleaving an Arg-Gly dipeptide from the β-NGF C-terminus (15, 37). In our experimental data, the lower amounts of Klk1b3 and other kallikrein 1b family members point to a reduced formation of the 7S NGF complex as a whole. Although the complex’s biological function has been reported to be of a competitive nature to NGF’s binding potential (38–40) by enclosing the active β-NGF in a horse-shoe structure (14), the reduced availability of the α and γ subunits would also leave β-NGF exposed to the proteolytic activity of Klk1b22, and at the same time prevent its C-terminal cleavage by the activity of γ-NGF (Klk1b3). Indeed, we have validated the high-throughput proteomic findings by western blot and by immunohistochemical visualization and confirmed that the increased abundance of Klk1b22 in knockout animals coincides with lower amounts of β-NGF both in males and in females, where in the latter, β-NGF is barely detectable. This inverse correlation also applied to the sub-cellular localization of β-NGF at least in male mice (where the β-NGF signal was visible): The Klk1b22 rich basal cell regions where negative for β-NGF signal, and β-NGF was only detected at the apical side.

The above observations led us to construct a working mechanistic model for the explanation of the observed SS-like phenotype in ERdj5 knockout animals (**Figure 6**). This model supposes an anti-inflammatory effect of NGF within the tissue in normal conditions, and attributes the increased immune responses and the subsequent inflammatory lesions to the lack of this inhibitory action, in conjunction with an initial auto-reactive stimulus that could be Klk1b22 itself or another autoantigen. The hypothesis that NGF exhibits an anti-inflammatory effect in the tissue is not without controversy, though. In one hand, a plethora of studies suggest that NGF indeed has anti-inflammatory actions, its blocking by antibodies can exacerbate inflammatory reactions (41) and it has been suggested and used as a treating agent (42). Inversely, there are several other studies which, in the serum, synovial fluid, cerebrospinal fluid and tissue samples of patients of various autoimmune diseases find levels of NGF that are either elevated compared to healthy individuals, or even correlated to disease activity (43). This apparent contradiction has been proposed to be due to the nature of NGF of both inciting immune responses, but also activating anti-inflammatory pathways to limit tissue damage (44). In the context of SS, knowledge on the involvement of NGF is limited to two studies in patient sera and one in cultured cells from patients. Serum levels of NGF have been found increased, but this was attributed mainly to its increased production from activated B-cells and chronic inflammation rather as driving force of inflammation (45). A different study did not find significant differences in the serum levels of NGF between patients and controls, but did associate NGF with T-cell activation and hypergammaglobulinaemia (46). Cultured epithelial cells from pSS patients were found to have elevated expression of both β-NGF and its TrkA receptor (47). The local activity of NGF in the salivary glands of SS patients has not yet been investigated.

In our model for the development of the SS-like phenotype in ERdj5 knockout mice, the upregulation of Klk1b22 can have three effects: It can directly reduce levels of β-NGF by cleavage, it may act as an autoantigen triggering immune responses and it also may be responsible for the limited transcription for all the other kallikreins, with which it shares adjacent loci (48). Regardless of the mechanism, the reduced transcription of other kallikreins that are components of the 7S NGF complex may result in its limited abundance in the tissue. This in turn results in reduced inhibitory potential against inflammatory reactions, leading to exacerbated and uncontrolled inflammation. Regarding the upstream triggers that can connect ERdj5 ablation to the increased Klk1b22 expression, it has been established that the absence of ERdj5 induces ER-stress in the murine salivary glands (9). In our proteomic data, heat shock cognate 71 kDa protein (Hspa8) is an ER-stress related molecular chaperone that stands out as significantly downregulated in both male and female knockout animals and was at the center of the ER-stress related STRING networks. This upstream intermediate link has not been explored deeper in this study, but it is a fertile ground for further investigation.

It is interesting that human kallikreins have been also found to differ between SS patients and healthy individuals in several previous studies. Also, in newer, high throughput proteomic and transcriptomic studies, various kallikreins, other proteases and proteins relevant to our results and working model have been found in significant deregulation in SS patients, although they were not the main focus of the specific studies. Specifically, the total kallikrein activity levels have been found elevated compared to healthy donors in SS patients (49, 50). Kallikrein levels in plasma and saliva were also found to be upregulated in SS patients using quantitative methods (51) and have been suggested as SS biomarkers (52). Even more, treatment with a kallikrein inhibitor has produced positive results in a limited number of chronic parotitis patients (53). In a proteomic study, human kallikreins 1, 6 and 11 have been found to have significantly different abundances in the saliva of SS patients, with KLK6 and KLK11 being upregulated in all patient groups *vs* healthy subjects, and KLK1 being downregulated in patients with high focus score and upregulated in patients with low focus score (54). Similarly, in a transcriptomic study, KLK6 was upregulated in cell lines derived from SS patients with high focus score compared to patients with low focus score (55). Overall, SS patient samples also presented a deregulation, compared to control samples, in various proteases like serpins, MMPs and their inhibitors (55). Another proteomic study found significantly elevated KLK14 in extracellular vesicles (EVs) isolated from whole saliva of non-SS subjects *vs*. pSS patients (56). The same study found multiple other deregulated proteins in the saliva or tears of SS patients that were either directly homolog or highly similar to significant proteins in our study as well (upregulated DnaJC3, Hspa1a, Annexin A1, Annexin A4 in SS patient EVs from whole saliva and Annexins A4, A6, A9 and Hsp74 in SS patient tear fluid). An earlier proteomic analysis of EVs from the same group had identified multiple proteasome subunit proteins, PDIs, Annexins, and Heat shock proteins deregulated in SS patients, as they were in our mouse model as well (57). A recent study of human minor salivary glands in female pSS patients also found multiple annexins with differential transcription regulation (A6 upregulated, A2, A3, A4, A5, A7 downregulated and the A2 receptor upregulated), deregulated HSPs, along with upregulated proteinases like MMP9 and various serpins (58). Also, a Weighted Gene Co-Expression Network Analysis identified the ER-stress signaling EIF2AK2 as a central hub protein in pathways relevant to SS (59). This complements our previous finding of elevated ER-stress in the salivary glands of SS patients due to increased XBP1s signaling (8), which seems to be a key factor to the disease development.

All the above published findings and our presented data validate the value of the ERdj5^-/-^ mouse model that exhibits highly similar reactions to the human disease and can highlight potential pathways of significance to SS that might have been overlooked. Moreover, the establishment of a solid mechanistic model that can describe a sequence of interactions leading to the inflammatory response in the ERdj5^-/-^ mouse will provide insights for further investigation towards the better understanding of the underlying mechanism in the fight against Sjögren’s syndrome.

## Data Availability Statement

The mass spectrometry proteomics data have been deposited to the ProteomeXchange Consortium *via* the PRIDE (60) partner repository with the dataset identifier PXD026424 (https://www.ebi.ac.uk/pride/archive/projects/PXD026424).

## Ethics Statement

The animal study was reviewed and approved by Animal Care and Use Committee, Veterinarian Administration, Attiki prefecture.

## Author Contributions

PM: Conceptualization, Methodology, Resources, Investigation, Formal analysis, Software, Data Curation, Validation, Visualization, Writing - original draft, Writing - review & editing. NY-F: Methodology, Investigation, Validation, Visualization. EA: Conceptualization, Resources, Writing - review & editing. AT: Conceptualization, Methodology, Writing - review & editing. MT: Methodology, Formal analysis, Software, Writing - review & editing. GS: Conceptualization, Methodology, Project administration, Formal analysis, Data Curation, Supervision, Funding acquisition, Writing - review & editing. All authors contributed to the article and approved the submitted version.

## Funding

This work was financed with grants from Linköping’s University (PM, NY, MT, and GS) and the State Scholarships Foundation, IKY, Greece (EA).

## Conflict of Interest

The authors declare that the research was conducted in the absence of any commercial or financial relationships that could be construed as a potential conflict of interest.

## Supplementary Material

Supplementary Image 1 Classification of proteins with significant proteomic relative abundance difference in the comparisons between wildtypes and knockouts according to KEGG pathways and reactome pathways in STRING analysis. Results for both sexes are presented. The proteins which were identified with significantly different relative abundances but were not part of any of the specific pathways are not presented.

Supplementary Image 2 Quantitative comparisons of all Kallikrein 1-related proteases in the murine submandibular salivary gland tissue that were detected and tested in this study but were not presented in a main figure, at the protein level (NSAF and emPAI mass spec quantifications of relative abundances) and at the transcription level (qRT-PRC comparative fold change amounts). Statistically significant differences between FWT *vs* FKO or MWT *vs* MKO groups are indicated as *p < 0.05, and **p < 0.01.

## References

[1] Kapsogeorgou EK Voulgarelis M Tzioufas AG. Predictive Markers of Lymphomagenesis in Sjögren’s Syndrome: From Clinical Data to Molecular Stratification. J Autoimmun (2019) 104:102316. 10.1016/j.jaut.2019.102316 31431317

[2] Jonsson R Bolstad AI Brokstad KA Brun JG. Sjögren’s Syndrome–a Plethora of Clinical and Immunological Phenotypes With a Complex Genetic Background. Ann N Y Acad Sci (2007) 1108:433–47. 10.1196/annals.1422.046 17894008

[3] Mitsias DI Kapsogeorgou EK Moutsopoulos HM. The Role of Epithelial Cells in the Initiation and Perpetuation of Autoimmune Lesions: Lessons From Sjogren’s Syndrome (Autoimmune Epithelitis). Lupus (2006) 15(5):255–61. 10.1191/0961203306lu2290rr 16761498

[4] Junjappa RP Patil P Bhattarai KR Kim HR Chae HJ. IRE1α Implications in Endoplasmic Reticulum Stress-Mediated Development and Pathogenesis of Autoimmune Diseases. Front Immunol (2018) 9:1289. 10.3389/fimmu.2018.01289 29928282 PMC5997832

[5] Sepúlveda D Barrera MJ Castro I Aguilera S Carvajal P Lagos C. Impaired IRE1α/XBP-1 Pathway Associated to DNA Methylation Might Contribute to Salivary Gland Dysfunction in Sjögren’s Syndrome Patients. Rheumatol (Oxford) (2018) 57(6):1021–32. 10.1093/rheumatology/key021 29534223

[6] Barrera MJ Aguilera S Castro I Cortés J Bahamondes V Quest AFG. Pro-Inflammatory Cytokines Enhance ERAD and ATF6α Pathway Activity in Salivary Glands of Sjögren’s Syndrome Patients. J Autoimmun (2016) 75:68–81. 10.1016/j.jaut.2016.07.006 27461470

[7] Katsiougiannis S Tenta R Skopouli FN. Endoplasmic Reticulum Stress Causes Autophagy and Apoptosis Leading to Cellular Redistribution of the Autoantigens Ro/Sjögren’s Syndrome-Related Antigen A (SSA) and La/SSB in Salivary Gland Epithelial Cells. Clin Exp Immunol (2015) 181(2):244–52. 10.1111/cei.12638 PMC4516440 25845745

[8] Apostolou E Moustardas P Iwawaki T Tzioufas AG Spyrou G. Ablation of the Chaperone Protein ERdj5 Results in a Sjögren’s Syndrome-Like Phenotype in Mice, Consistent With an Upregulated Unfolded Protein Response in Human Patients. Front Immunol (2019) 10:506. 10.3389/fimmu.2019.00506 30967862 PMC6438897

[9] Hosoda A Tokuda M Akai R Kohno K Iwawaki T. Positive Contribution of ERdj5/JPDI to Endoplasmic Reticulum Protein Quality Control in the Salivary Gland. Biochem J (2009) 425(1):117–25. 10.1042/bj20091269 19788412

[10] Todd DJ Lee AH Glimcher LH. The Endoplasmic Reticulum Stress Response in Immunity and Autoimmunity. Nat Rev Immunol (2008) 8(9):663–74. 10.1038/nri2359 18670423

[11] Clements JA Willemsen NM Myers SA Dong Y. The Tissue Kallikrein Family of Serine Proteases: Functional Roles in Human Disease and Potential as Clinical Biomarkers. Crit Rev Clin Lab Sci (2004) 41(3):265–312. 10.1080/10408360490471931 15307634

[12] Linker R Gold R Luhder F. Function of Neurotrophic Factors Beyond the Nervous System: Inflammation and Autoimmune Demyelination. Crit Rev Immunol (2009) 29(1):43–68. 10.1615/critrevimmunol.v29.i1.20 19348610

[13] Fahnestock M Woo JE Lopez GA Snow J Walz DA Arici MJ. beta-NGF-endopeptidase: Structure and Activity of a Kallikrein Encoded by the Gene mGK-22. Biochemistry (1991) 30(14):3443–50. 10.1021/bi00228a014 2012805

[14] Bax B Blundell TL Murray-Rust J McDonald NQ. Structure of Mouse 7S NGF: A Complex of Nerve Growth Factor With Four Binding Proteins. Structure (1997) 5(10):1275–85. 10.1016/s0969-2126(97)00280-3 9351801

[15] Uddin M Beg OU. Cleavage Specificities of Individual Members of Kallikrein Family of Proteins on Synthetic Peptide Containing the Bradykinin Sequence. J Protein Chem (1998) 17(3):291–4. 10.1023/a:1022597004834 9588954

[16] Isackson PJ Ullrich A Bradshaw RA. Mouse 7S Nerve Growth Factor: Complete Sequence of a cDNA Coding for the Alpha-Subunit Precursor and Its Relationship to Serine Proteases. Biochemistry (1984) 23(25):5997–6002. 10.1021/bi00320a015 6395888

[17] Barker PA Murphy RA. The Nerve Growth Factor Receptor: A Multicomponent System That Mediates the Actions of the Neurotrophin Family of Proteins. Mol Cell Biochem (1992) 110(1):1–15. 10.1007/bf02385000 1315923

[18] Vega JA García-Suárez O Hannestad J Pérez-Pérez M Germanà A. Neurotrophins and the Immune System. J Anat (2003) 203(1):1–19. 10.1046/j.1469-7580.2003.00203.x 12892403 PMC1571144

[19] Wiśniewski JR Zougman A Nagaraj N Mann M. Universal Sample Preparation Method for Proteome Analysis. Nat Methods (2009) 6(5):359–62. 10.1038/nmeth.1322 19377485

[20] Tatsumi K Ohashi K Taminishi S Okano T Yoshioka A Shima M. Reference Gene Selection for Real-Time RT-PCR in Regenerating Mouse Livers. Biochem Biophys Res Commun (2008) 374(1):106–10. 10.1016/j.bbrc.2008.06.103 18602371

[21] Szklarczyk D Gable AL Lyon D Junge A Wyder S Huerta-Cepas J. String v11: Protein-Protein Association Networks With Increased Coverage, Supporting Functional Discovery in Genome-Wide Experimental Datasets. Nucleic Acids Res (2019) 47(D1):D607–d13. 10.1093/nar/gky1131 PMC6323986 30476243

[22] Yamamoto M Nakata H Kumchantuek T Adhapanyawanich K Iseki S. Distinct Hormonal Regulation of Two Types of Sexual Dimorphism in Submandibular Gland of Mice. Cell Tissue Res (2018) 371(2):261–72. 10.1007/s00441-017-2719-4 29079883

[23] Treister NS Richards SM Lombardi MJ Rowley P Jensen RV Sullivan DA. Sex-Related Differences in Gene Expression in Salivary Glands of BALB/c Mice. J Dent Res (2005) 84(2):160–5. 10.1177/154405910508400210 15668334

[24] Mukaibo T Gao X Yang NY Oei MS Nakamoto T Melvin JE. Sexual Dimorphisms in the Transcriptomes of Murine Salivary Glands. FEBS Open Bio (2019) 9(5):947–58. 10.1002/2211-5463.12625 PMC6487692 30998297

[25] Stopka P Kuntová B Klempt P Havrdová L Černá M Stopková R. On the Saliva Proteome of the Eastern European House Mouse (Mus Musculus Musculus) Focusing on Sexual Signalling and Immunity. Sci Rep (2016) 6:32481. 10.1038/srep32481 27577013 PMC5006050

[26] Mavragani CP Moutsopoulos HM. Sjögren’s Syndrome: Old and New Therapeutic Targets. J Autoimmun (2020) 110:102364. 10.1016/j.jaut.2019.102364 31831255

[27] Michael D Soi S Cabera-Perez J Weller M Alexander S Alevizos I. Microarray Analysis of Sexually Dimorphic Gene Expression in Human Minor Salivary Glands. Oral Dis (2011) 17(7):653–61. 10.1111/j.1601-0825.2011.01816.x PMC4083356 21819492

[28] Hu ZQ Murakami K Ikigai H Shimamura T. Enhancement of Lymphocyte Proliferation by Mouse Glandular Kallikrein. Immunol Lett (1992) 32(1):85–9. 10.1016/0165-2478(92)90204-2 1500088

[29] Hayashi Y Hirokawa K. Immunopathology of Experimental Autoallergic Sialadenitis in C3H/He Mice. Clin Exp Immunol (1989) 75(3):471–6. PMC1541949 2784749

[30] Liu SH Zhou DH Hess AD. Adoptive Transfer of Experimental Autoimmune Dacryoadenitis in Susceptible and Resistant Mice. Cell Immunol (1993) 150(2):311–20. 10.1006/cimm.1993.1199 8370074

[31] Liu SH Zhou DH. Experimental Autoimmune Dacryoadenitis: Purification and Characterization of a Lacrimal Gland Antigen. Invest Ophthalmol Vis Sci (1992) 33(6):2029–36. 1582807

[32] Liu SH Prendergast RA Silverstein AM. Experimental Autoimmune Dacryoadenitis. I. Lacrimal Gland Disease in the Rat. Invest Ophthalmol Vis Sci (1987) 28(2):270–5. 8591907

[33] Jiang G Ke Y Sun D Li H Ihnen M Jumblatt MM. A New Model of Experimental Autoimmune Keratoconjunctivitis Sicca (KCS) Induced in Lewis Rat by the Autoantigen Klk1b22. Invest Ophthalmol Vis Sci (2009) 50(5):2245–54. 10.1167/iovs.08-1949 19060269

[34] Wu C Wang Z Zourelias L Thakker H Passineau MJ. IL-17 Sequestration *Via* Salivary Gland Gene Therapy in a Mouse Model of Sjogren’s Syndrome Suppresses Disease-Associated Expression of the Putative Autoantigen Klk1b22. Arthritis Res Ther (2015) 17(1):198. 10.1186/s13075-015-0714-2 26245278 PMC4527205

[35] Woo SB Timm DE Neet KE. Alteration of NH2-terminal Residues of Nerve Growth Factor Affects Activity and Trk Binding Without Affecting Stability or Conformation. J Biol Chem (1995) 270(11):6278–85. 10.1074/jbc.270.11.6278 7890765

[36] Prencipe G Minnone G Strippoli R De Pasquale L Petrini S Caiello I. Nerve Growth Factor Downregulates Inflammatory Response in Human Monocytes Through TrkA. J Immunol (2014) 192(7):3345–54. 10.4049/jimmunol.1300825 24585880

[37] Fahnestock M. Structure and Biosynthesis of Nerve Growth Factor. Curr Top Microbiol Immunol (1991) 165:1–26. 10.1007/978-3-642-75747-1_1 2032463

[38] Woodruff NR Neet KE. Inhibition of Beta Nerve Growth Factor Binding to PC12 Cells by Alpha Nerve Growth Factor and Gamma Nerve Growth Factor. Biochemistry (1986) 25(24):7967–74. 10.1021/bi00372a027 3026466

[39] Stach RW Shooter EM. Cross-Linked 7S Nerve Growth Factor Is Biologically Inactive. J Neurochem (1980) 34(6):1499–505. 10.1111/j.1471-4159.1980.tb11230.x 7381472

[40] Harris-Warrick RM Bothwell MA Shooter EM. Subunit Interactions Inhibit the Binding of Beta Nerve Growth Factor to Receptors on Embryonic Chick Sensory Neurons. J Biol Chem (1980) 255(23):11284–9. 10.1016/S0021-9258(19)70288-9 6254965

[41] Reinshagen M Rohm H Steinkamp M Lieb K Geerling I Von Herbay A. Protective Role of Neurotrophins in Experimental Inflammation of the Rat Gut. Gastroenterology (2000) 119(2):368–76. 10.1053/gast.2000.9307 10930372

[42] Lambiase A Bonini S Aloe L Rama P Bonini S. Anti-Inflammatory and Healing Properties of Nerve Growth Factor in Immune Corneal Ulcers With Stromal Melting. Arch Ophthalmol (2000) 118(10):1446–9. 10.1001/archopht.118.10.1446 11030834

[43] Seidel MF Herguijuela M Forkert R Otten U. Nerve Growth Factor in Rheumatic Diseases. Semin Arthritis Rheum (2010) 40(2):109–26. 10.1016/j.semarthrit.2009.03.002 19481238

[44] Minnone G De Benedetti F Bracci-Laudiero L. NGF and Its Receptors in the Regulation of Inflammatory Response. Int J Mol Sci (2017) 18(5):1028. 10.3390/ijms18051028 PMC5454940 28492466

[45] Li YJ Yang CS Lei L Wu KF Yang PT Xiao WG. Serum Nerve Grow Factor and Brain-Derived Neurotrophic Factor Profiles in Sjögren’s Syndrome Concomitant With Interstitial Lung Disease. Clin Rheumatol (2014) 33(8):1161–4. 10.1007/s10067-014-2588-0 24691584

[46] Fauchais AL Boumediene A Lalloue F Gondran G Loustaud-Ratti V Vidal E. Brain-Derived Neurotrophic Factor and Nerve Growth Factor Correlate With T-Cell Activation in Primary Sjogren’s Syndrome. Scand J Rheumatol (2009) 38(1):50–7. 10.1080/03009740802378832 18830907

[47] Lisi S Sisto M Ribatti D D’Amore M De Lucro R Frassanito MA. Chronic Inflammation Enhances NGF-β/TrkA System Expression *Via* EGFR/MEK/ERK Pathway Activation in Sjögren’s Syndrome. J Mol Med (Berl) (2014) 92(5):523–37. 10.1007/s00109-014-1130-9 24557415

[48] Olsson AY Lundwall A. Organization and Evolution of the Glandular Kallikrein Locus in Mus Musculus. Biochem Biophys Res Commun (2002) 299(2):305–11. 10.1016/s0006-291x(02)02629-3 12437987

[49] Friberg B Jonsson R Linde A. Salivary Kallikrein in Sjögren’s Syndrome. Clin Exp Rheumatol (1988) 6(2):135–8. 3180538

[50] Beeley JA Khoo KS. Salivary Proteins in Rheumatoid Arthritis and Sjögren’s Syndrome: One-Dimensional and Two-Dimensional Electrophoretic Studies. Electrophoresis (1999) 20(7):1652–60. 10.1002/(sici)1522-2683(19990601)20:7<1652::Aid-elps1652>3.0.Co;2-r 10424492

[51] Hernández CC Donadi EA Reis ML. Kininogen-Kallikrein-Kinin System in Plasma and Saliva of Patients With Sjögren’s Syndrome. J Rheumatol (1998) 25(12):2381–4. 9858433

[52] Baldini C Ferro F Elefante E Bombardieri S. Biomarkers for Sjögren’s Syndrome. Biomark Med (2018) 12(3):275–86. 10.2217/bmm-2017-0297 29460647

[53] Maier H Adler D Lenarz T Müller-Esterl W. New Concepts in the Treatment of Chronic Recurrent Parotitis. Arch Otorhinolaryngol (1985) 242(3):321–8. 10.1007/bf00453557 2416305

[54] Cecchettini A Finamore F Ucciferri N Donati V Mattii L Polizzi E. Phenotyping Multiple Subsets in Sjögren’s Syndrome: A Salivary Proteomic SWATH-MS Approach Towards Precision Medicine. Clin Proteomics (2019) 16:26. 10.1186/s12014-019-9245-1 31249499 PMC6587286

[55] Vakrakou AG Polyzos A Kapsogeorgou EK Thanos D Manoussakis MN. Perturbation of Transcriptome in Non-Neoplastic Salivary Gland Epithelial Cell Lines Derived From Patients With Primary Sjögren’s Syndrome. Data Brief (2018) 17:194–9. 10.1016/j.dib.2017.12.023 PMC5988450 29876386

[56] Aqrawi LA Galtung HK Guerreiro EM Øvstebø R Thiede B Utheim TP. Proteomic and Histopathological Characterisation of Sicca Subjects and Primary Sjögren’s Syndrome Patients Reveals Promising Tear, Saliva and Extracellular Vesicle Disease Biomarkers. Arthritis Res Ther (2019) 21(1):181. 10.1186/s13075-019-1961-4 31366407 PMC6670195

[57] Aqrawi LA Galtung HK Vestad B Øvstebø R Thiede B Rusthen S. Identification of Potential Saliva and Tear Biomarkers in Primary Sjögren’s Syndrome, Utilising the Extraction of Extracellular Vesicles and Proteomics Analysis. Arthritis Res Ther (2017) 19(1):14. 10.1186/s13075-017-1228-x 28122643 PMC5264463

[58] Oyelakin A Horeth E Song E-AC Min S Che M Marzullo B. Transcriptomic and Network Analysis of Minor Salivary Glands of Patients With Primary Sjögren’s Syndrome. Front Immunol (2021) 11(606268):3352. 10.3389/fimmu.2020.606268 PMC7821166 33488608

[59] Yao Q Song Z Wang B Qin Q Zhang JA. Identifying Key Genes and Functionally Enriched Pathways in Sjögren’s Syndrome by Weighted Gene Co-Expression Network Analysis. Front Genet (2019) 10:1142. 10.3389/fgene.2019.01142 31798636 PMC6863930

[60] Perez-Riverol Y Csordas A Bai J Bernal-Llinares M Hewapathirana S Kundu DJ. The PRIDE Database and Related Tools and Resources in 2019: Improving Support for Quantification Data. Nucleic Acids Res (2019) 47(D1):D442–D50. 10.1093/nar/gky1106 PMC6323896 30395289

