# Supplementary material for: Deregulation of the Kallikrein Protease Family in the Salivary Glands of the Sjögren’s Syndrome ERdj5 Knockout Mouse Model
Source: Front Immunol. 2021 Jul 7;12:693911. doi: 10.3389/fimmu.2021.693911 (PMC8292930; doi:10.3389/fimmu.2021.693911)
Supplement: Supplementary Image 1 — Classification of proteins with significant proteomic relative abundance difference in the comparisons between wildtypes and knockouts according to KEGG pathways and reactome pathways in STRING analysis. Results for both sexes are presented. The proteins which were identified with significantly different relative abundances but were not part of any of the specific pathways are not presented. [file DataSheet_1.zip › 693911_SupMaterial/Supplementary Methods.DOCX]

Supplementary Methods

Experimental samples

Tissue samples of whole submandibular salivary glands from mice were obtained after the experimental animals were sacrificed by blood draining via the inferior vena cava under ketamine/xylazine intraperitoneal anesthesia (anesthetic solution of 20mg/mL ketamine, 1.33mg/mL xylazine in H_2_O; 170mg ketamine and 11.33mg xylazine per kg of animal weight). Tissues were washed in PBS pH=7.4 and cleaned from the surrounding fat and connective tissue with micro-surgical scissors and appropriately maintained for the subsequent experiments. The right submandibular glands were snap frozen by immersion in dry-ice cold isopentane and are the samples used in this study for mass spectrometry, qRT-PCR and western blotting. The left submandibular glands were fixed in a 10% formalin solution (4% formaldehyde, 1% methanol) at 4^o^C overnight. After fixation they were washed with tap water and processed with standard histology methods (dehydrated in ascending concentrations of 70%, 80%, 95% and 2x 100% ethanol), cleared twice in xylene, and embedded by 2x immersion in 60^o^C paraffin. They were afterwards molded into blocks of paraffin wax. Paraffin blocks were sectioned at 3μm thickness in a Leica RM2125 RTS microtome and are the samples used in this study for histochemical and immunohistochemical imaging.

Histological examination of mouse salivary glands

Paraffin-embedded sections of salivary glands from the mice of all experimental groups were deparaffinized by immersing in xylene, followed by dehydration in descending concentrations of ethanol (2x 100%, 95%, 80%, 50%) and finally immersion in tap water. The prepared tissue sections were stained with Harris hematoxylin and eosin (H&E) (1min immersion in hematoxylin stain, wash with tap water x3, 1min immersion in Eosin stain), dehydrated in ascending concentrations of ethanol and xylene (80%, 95%, 2x 100% EtOH – 2x xylene) and mounted with DPX. Stained sections were observed at 200x magnification in a microscope and digital images were captured.

Sample preparation for proteomic analysis^1^

While still frozen at -80^o^C, a small piece (18-30 mg) of salivary gland tissue was cut with a dry-ice cold scalpel from the lower tip of the gland. The cut piece was transferred in a 1.5 mL microcentrifuge tube and placed on dry ice. Then the scalpel was cleaned two times with ddH2O to avoid cross contamination and the process repeated for all samples while on dry ice. The samples were weighted and then pulverized while frozen with a plastic centrifuge tube micropestle (one micropestle per sample). 150 μL of cold lysis buffer (0.1M Tris-HCl pH 7.6, 4% SDS and 0.1M DTE) were added to each tube and the samples where homogenized using the same micropestle and rapid mechanical rotation. Afterwards, samples were cold bath sonicated for 10 min. After homogenization, samples were centrifuged at 13000 RPM (rotor 75003424: 13200G – 15100G) for 10 min at RT. The supernatant was transferred to a new tube, and a 1 μL sample was diluted 1:20 and used to measure the protein concentration with the Bradford colorimetric assay (Bio-Rad [#5000006](https://www.bio-rad.com/en-se/sku/5000006-bio-rad-protein-assay-dye-reagent-concentrate-450-ml?ID=5000006)). For each sample, the appropriate volume for 200 μg total protein was calculated (20-50 μL) and mixed with 200 μL Urea buffer (8M Urea in 0.1M Tris-HCl pH 8.5) in the filter unit of an Amicon Ultra 0.5 mL, 30 kDa cutoff filter-microtube assembly (Merck-millipore [#UFC500396](https://www.sigmaaldrich.com/catalog/product/mm/ufc5003)). The samples were centrifuged at 13000 RPM at RT for 15 min. This centrifugation step was repeated by adding 200μL to the remaining sample in the filter unit and further centrifugation for 15 min at RT. Afterwards, the flow-through from the two centrifugations was discarded and 100 μL of Iodoacetamide solution were added (0.05 M IAM in Urea buffer) in the remaining fluid in the filter unit, mixed by pipetting and incubated for 20 min at RT in the dark. The units were centrifuged once again at 13000 RPM for 10 min. Two steps of 100 μL Urea buffer addition in the filter unit and 13000 RPM centrifugation for 15 min at RT were performed, and the flowthrough was discarded when necessary in order not to fill the collection tube. In order to perform a buffer exchange, two steps of 100 μL ABC buffer (50 mM NH_4_HCO_3_ pH 8 in MilliQ H_2_O) addition to the fluid remaining in the filter unit and subsequent 13000 RPM centrifugation for 10 min were carried out. After that, the filter units were transferred to new, clean collection tubes and 40 μL of ABC buffer with 500 ng/μL trypsin solution were added to the remaining fluid. Samples were incubated in a humidified container overnight at RT and in the dark. Next, the peptide-containing filtrate was collected by centrifugation at 13000 RPM for 10 min. Without throwing away the flowthrough, centrifugation was repeated after the addition of 40 μL of ABC buffer in the filter unit. The combined flowthrough liquid containing the collected peptides was frozen at -80^o^C. The lids of the tubes were opened, and the tubes were sealed with double parafilm, to which small holes were punctured with a needle, while keeping the samples frozen. The frozen samples were lyophilized overnight using vacuum lyophilizer set at -80^o^C. Dried samples were stored at ‑20^o^C until the LC-MS/MS analysis.

Liquid chromatography- tandem mass spectrometry (LC-MS/MS)

The lyophilized samples were reconstituted in 10 µL 0.1% formic acid (v/v) in MilliQ H_2_O and separated by reverse phase chromatography. 4 µL of reconstituted peptides were injected into a reverse-phase 20 mm × 100 μm C18 pre-column followed by a 100 mm × 75 μm C18 column with particle size 5 μm (NanoSeparations, Nieuwkoop, Netherlands) at a flow rate of 300 nL/min on EASY-nLC II (Thermo Scientific) by a gradient of 0.1% formic acid in MilliQ H_2_O (A) and 0.1% formic acid in acetonitrile (B) as follows: From 2% B to 30% B in 70 min; from 30% B to 100% B in 45 min. Automated online analyses were performed in positive mode by LTQ Orbitrap Velos Pro hybrid mass spectrometer (Thermo Scientific) equipped with a nano-electrospray source with Xcalibur software (v.2.6, Thermo Scientific). Full MS scans were collected with a range of 350–1800 *m*/*z*, a resolution of 30
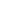
000 (*m*/*z* 200), the top 20 most intense multiple charged ions were selected with an isolation window of 2.0 and fragmented in the linear ion trap by collision-induced dissociation with normalized collision energy of 30%. Dynamic exclusion was enabled ensuring peaks selected for fragmentation were excluded for 60 s.

Protein identification

The generated raw files were analysed using Sequest HT in Proteome Discoverer (Thermo Fisher, San Jose, CS version 1.4.0.288) and the *Mus musculus* protein database from Uniprot (uniprot.org). The following search parameters were used: trypsin as a digestion enzyme; maximum number of missed cleavages 2; fragment ion mass tolerance 0.60 Da; parent ion mass tolerance 10.0 ppm; carbamidomethylation of cysteine as fixed modification.

Data evaluation and relative protein quantification

Identified proteins were validated using SCAFFOLD software (Version 4.5.3; Proteome Software Inc., Portland, OR). Identifications were based on a minimum of 2 peptides, minimum 80% peptide identification probability (using the Scaffold Local FDR algorithm), and minimum 95% protein identification probability using the Protein Prophet algorithm^2^. Proteins, which contained similar peptides, and which could not be differentiated based on LC-MS/MS analysis alone were grouped to satisfy the principles of parsimony.

The relative abundances of the identified proteins were estimated using the normalized total spectra counts, the exponentially modified protein abundance index (emPAI) and the normalized spectral abundance factor (NSAF) in Scaffold. Quantitative differences were statistically analyzed by Student’s *t*-test with the Benjamini-Hochberg correction. Differences with *p*-values ≤ 0.05 were considered statistically significant.

The group pairs for which protein abundance comparisons were performed where the female wildtype mice vs the male wildtype mice (FWT vs MWT), the female wildtype mice vs the female ERd5^-/-^ mice (FWT vs FKO) and the male wildtype mice vs the male ERd5^-/-^ mice (MWT vs MKO). Additionally, proteins that were found to differ significantly between wild type animals and knockouts in both sexes were compared using a 2-way ANOVA analysis and *p*-values ≤ 0.05 were considered statistically significant.

qRT-PCR - Primer Sequences

While still frozen at -80^o^C, a small piece of size in the range of 20mg of salivary gland tissue was cut with a dry-ice cold scalpel from the side that was previously cut for protein extraction. Total RNA was isolated from the salivary glands of the same animals whose tissue samples were used for the proteomic analysis, using Fisher BioReagents™ SurePrep™ Purification Kit (Fisher Scientific [#BP280250](https://www.fishersci.com/shop/products/fisher-bioreagents-sureprep-rna-dna-protein-purification-kit-2/bp280250)) according to the manufacturer’s protocol. First strand cDNA was synthesized from the total RNA using the iScript™ cDNA Synthesis Kit (Bio-Rad [#1708891](https://www.bio-rad.com/en-se/sku/1708891-iscript-cdna-synthesis-kit-100-x-20-ul-rxns?ID=1708891)). qRT-PCR was performed in duplicates using iTaq™ Universal SYBR® Green Supermix (Bio-Rad [#1725121](https://www.bio-rad.com/en-se/sku/1725121-itaq-universal-sybr-green-supermix-500-x-20-ul-rxns-5-ml-5-x-1-ml?ID=1725121)) with either the Applied Biosystems^TM^ 7500 or Applied Biosystems^TM^7900 HT Fast Real-Time PCR system (Thermo Fisher [#4351104](https://www.thermofisher.com/order/catalog/product/4351104#/4351104), [#4329001](https://www.thermofisher.com/order/catalog/product/4329001#/4329001)). Primer sequences for amplification were either obtained from PrimerBank^3^ or designed using Primer3^4^. The table below lists all primers that were used and their sources.

Primer sequences used for kallikreins and housekeeping gene

| **Gene** | **PrimerBank ID^3^** |  | **Sequence (5' -> 3')** |
| --- | --- | --- | --- |
| Klk1b1 | 8659568a1 | FW | GGGGCAGCATTATACCTGTCA |
|  |  | RV | CCCTTCACTCCTGCACACA |
|  |  |  |  |
| Klk1b3 | 53374a1 | FW | TGACGACAACTACAAGGTTTGG |
|  |  | RV | GGAATCGGATGTGCTTCCTCAT |
|  |  |  |  |
| Klk1b4 | 8659572a1 | FW | GCACTACACCCATCAAGTTCA |
|  |  | RV | TCCTGAGTCATGCTCACAAGT |
|  |  |  |  |
| Klk1b5 | 7949064a1 | FW | CACCCGTCATATACGAACCCG |
|  |  | RV | AGTTTGGTGTAGATACCTGGCA |
|  |  |  |  |
| Klk1b8 | 7705572a1 | FW | AGCTCCTGCCTATTAAGAACTGT |
|  |  | RV | AGGGCCTGTTGATGTGATACC |
|  |  |  |  |
| Klk1b9 | 21426851a1 | FW | CAACCTATACGAAGAGGAACCCT |
|  |  | RV | AGGATGTCGGATGTGGTTTCTA |
|  |  |  |  |
| Klk1b11 | 28875780a1 | FW | CAGCACCGAATGGTCAGCA |
|  |  | RV | CTGGGGTTTGGAATTTCGTGG |
|  |  |  |  |
| Klk1b16 | 7019443a1 | FW | CTTTGAAAAGTTACCTCCTGGGG |
|  |  | RV | AGCTTGGTGAACATACACTGAAG |
|  |  |  |  |
| Klk1b21 | 23956056a1 | FW | ATGACCTGATGTTACTACGCCT |
|  |  | RV | GCAGGGGCTTGATGAACCC |
|  |  |  |  |
| Klk1b22 | 387167a1 | FW | TCAAGCTCCATCCTAATGAGGT |
|  |  | RV | CGGTGCATTGGGTTCACCA |
|  |  |  |  |
| Klk1b24 | 8393675a1 | FW | TGGCAAAAGCCAAATGACCTT |
|  |  | RV | ATTGGGTTTACCACATGGGAC |
|  |  |  |  |
| Klk1b26 | Designed via primer3^4^ | FW | AACTCGAACAGCTCCAGGAA |
|  |  | RV | CTGTCCTGTTGACACCCAGA |
|  |  |  |  |
| Klk1b27 | 12313877a1 | FW | CTGTGCTCCGCTCCAACAAA |
|  |  | RV | GTGGGATGTGGTCATTCAGGA |
|  |  |  |  |
| PPIA | 6679438c1 | FW | GAGCTGTTTGCAGACAAAGTTC |
|  |  | RV | CCCTGGCACATGAATCCTGG |

Immunohistochemistry

Paraffin-embedded tissues of the mouse salivary glands were sectioned and mounted onto microscope slides. Slides were deparaffinized and hydrated (Immersion in xylene 2x 5min, EtOH 100% 2x 5min, EtOH 95% 1x 2min, EtOH 80% 1x 2min, EtOH 50% 1x 2min, tap water). Antigen retrieval was performed in the PT Link Pre-Treatment Module at 60^o^C t 95^o^C for 20 min 🡪 60^o^C with the DAKO citrate target retrieval solution, pH=6.1. After retrieval slides were washed in 2x tap water. Endogenous peroxidase activity was blocked by immersion of the slides in 0.5% H_2_O_2_ in methanol for 20min in the dark. After washing 3x in tap water, the tissues on the slides were inscribed with a PAP pen and non-immune fetal bovine serum (FBS) was used to block non-specific antibody binding: Sections incubated one hour with 100μL per section of blocking solution containing 10% FBS 0.1% Tween®20 in PBS for 1 hour. Sections were drained from the blocking solution and incubated with 50mL of primary antibody solutions (see antibody paragraph) per section overnight at 4°C in a humidified chamber. The next day, the slides were washed 3x with PBS followed by a two-hour incubation with appropriate secondary antibodies (see antibody paragraph). Afterwards, she slides were washed 3x in PBS and drained. After development with DAB (1 drop per section of Pierce™ DAB Substrate Kit solution -Thermo Scientific [#34002](https://www.thermofisher.com/order/catalog/product/34002#/34002)) for 10 min, sections were washed 2x with PBS, 1x in tap water, counterstained with Harris hematoxylin solution (10sec dips), washed 3x in tap water, dehydrated (10 sec dips in EtOH 70% 🡪 EtOH 80% 🡪 EtOH 95% 🡪 2x EtOH 100% 🡪 2x xylene), mounted with DPX and coversliped. Stained sections were visualized at 200x magnification on an Olympus microscope and digitally photographed.

Protein extracts and Western Blot

While still frozen at -80^o^C, a small piece of 18-30 mg of salivary gland tissue was cut with a dry-ice cold scalpel from the same tissue sample that was previously cut for MS sample preparation. The cut piece was transferred in a 1.5 mL microcentrifuge tube and placed on dry ice. Then the scalpel was cleaned two times with ddH2O to avoid cross contamination and the process repeated for all samples while on dry ice. The samples were weighted and then pulverized while frozen with a plastic centrifuge tube micropestle (one micropestle per sample). 150 μL per sample of cold RIPA lysis buffer supplemented with 10% protease inhibitor cocktail (cOmplete™ Mini Protease Inhibitor Cocktail from Merck [#11836153001 Roche](https://www.sigmaaldrich.com/catalog/product/roche/11836153001)) and 1mM PMSF (10 μL of 100mM PMSF in isopropanol per 1 mL final lysis solution) were added to each tube and the samples where homogenized using the same micropestle and rapid mechanical rotation and up and down pressure. Afterwards, samples were cold bath sonicated for 10 min. After homogenization, samples were centrifuged at 13000 RPM (rotor 75003424: 13200G – 15100G) for 10 min at RT. The supernatant was transferred to a new tube, and a small (1 μL) sample was diluted 1:20 and used to measure the protein concentration using the Pierce™ Microplate BCA Protein Assay Kit (Thermo Fisher [#23252](https://www.thermofisher.com/order/catalog/product/23252#/23252)). Appropriate volumes of each homogenate for 30 μg of total protein were prepared for BIS-polyacrylamide gel electrophoresis by mixing them in Laemmli Sample Buffer (Bio-Rad [#1610747](https://www.bio-rad.com/en-se/sku/1610747-4x-laemmli-sample-buffer?ID=1610747)), which had been diluted from 4x to 1x with ddH2O and supplemented with 1 mL of mercaptethanol for a total sample volume of 30 μL. Samples were boiled at 100^o^C in a heat plate for 10 min, then cooled and loaded in a 10 well 4–15% Mini-PROTEAN® TGX™ Precast Protein Gel (Bio-Rad [#4561084](https://www.bio-rad.com/en-se/sku/4561084-4-15-mini-protean-tgx-precast-protein-gels-10-well-50-ul?ID=4561084)), along with a dedicated well for 6 μL 10–250 kD Precision Plus Protein™ WesternC™ Protein Standard (Bio-Rad [#1610376](https://www.bio-rad.com/en-se/sku/1610376-precision-plus-protein-westernc-blotting-standards-250-ul?ID=1610376)). Gels were run in a Tris-glycine buffer (Bio-Rad [#1610771EDU](https://www.bio-rad.com/en-se/sku/1610771edu-10x-tris-glycine-buffer?ID=1610771edu)) in a vertical electrophoresis chamber (Bio-Rad [#1658004](https://www.bio-rad.com/en-se/sku/1658004-mini-protean-tetra-vertical-electrophoresis-cell-for-mini-precast-gels-4-gel?ID=1658004)) for 1 hour at a constant voltage of 100 V (34-19 mA). The proteins in the gel were transferred in a PVDF membrane using Trans-Blot Turbo Mini 0.2 µm PVDF Transfer Packs (Bio-Rad [#1704156](https://www.bio-rad.com/en-se/sku/1704156-trans-blot-turbo-mini-0-2-um-pvdf-transfer-packs?ID=1704156)) in a Trans-Blot® Turbo™ Transfer System (Bio-Rad [#1704150](https://www.bio-rad.com/en-se/sku/1704150-trans-blot-turbo-transfer-system?ID=1704150)) at a constant 2.0A with 25V limit for 7 min. The membranes with the transferred proteins were washed 3x in 0.1% Tween®20 in TBS in a shaker, sealed in plastic bags with 10mL of primary antibody solution (see antibodies paragraph) in a shaker at 4^o^C overnight. The next day, the membranes were washed 3x with 0.1% Tween®20 in TBS in a shaker, and then incubated for 2h with the secondary antibody solution (see antibodies paragraph). Lastly the membranes were washed 3x with 0.1% Tween®20 in TBS in a shaker, drained and placed on the photographing platform of a ChemiDoc XRS+ chamber (Bio-Rad [#1708265](https://www.bio-rad.com/en-se/sku/1708265-chemidoc-xrs-system-with-image-lab-software?ID=1708265)) and protein bands were visualized and photographed after spreading 1mL of Pierce™ ECL Western Blotting Substrate (Thermo Fisher [#32106](https://www.thermofisher.com/order/catalog/product/32106#/32106)) and covering them with a transparent membrane for isoform distribution of the substrate solution and bubble removal.

Antibodies and dilutions for Immunohistochemisrty and Western Blot

- Anti-Klk1b22: Rabbit Polyclonal IgG anti-mouse Klk1b22 ([orb355387](https://www.biorbyt.com/klk1b22-antibody-orb355387.html)), from biorbyt.

Dilution for IHC: 1:500 in 5% FBS 0.05% Tween20 in PBS

Dilution for WB: 1:1000 in 1% milk 0.1% Tween20 in TBS

- Anti-NGF: (for WB) Rabbit polyclonal IgG anti-mouse beta NGF ([AS13 2692](https://www.agrisera.com/shop?funk=visa_artikel&artnr=AS13%202692)), from Agrisera.

Dilution for WB: 1:1000 in 1% milk 0.1% Tween20 in TBS

- Anti-NGF: (for IHC) Rabbit polyclonal anti-human NGF ([E-AB-32239](https://www.elabscience.com/p-ngf_polyclonal_antibody-28564.html)), from elabscience.

Dilution for IHC: 1:400 in 5% FBS 0.05% Tween20 in PBS

- Anti-GAPDH: Mouse monoclonal anti-rabbit Glyceraldehyde-3-phosphate dehydrogenase [clone 6C5] ([MAB374](https://www.merckmillipore.com/SE/en/product/Anti-Glyceraldehyde-3-Phosphate-Dehydrogenase-Antibody-clone-6C5,MM_NF-MAB374?cid=BI-XX-BRC-A-NANT-ANTI-B096-1308)), from Merck-Millipore.

Dilution for WB: 1:2000 in 1% milk 0.1% Tween20 in TBS

- Anti-rabbit secondary HRP: Goat Anti-rabbit IgG (heavy and light chain), HRP-linked ([#7074](https://www.cellsignal.com/products/secondary-antibodies/anti-rabbit-igg-hrp-linked-antibody/7074)), from Cell Signaling Technology via Bionordica.

Dilution for IHC: 1:450 in 5% FBS 0.05% Tween20 in PBS

Dilution for WB: 1:2000 in 1% milk 0.1% Tween20 in TBS

- Anti-mouse secondary HRP: Horse anti-mouse IgG (heavy and light chain), conjugated to HRP ([#7076](https://www.cellsignal.com/products/secondary-antibodies/anti-mouse-igg-hrp-linked-antibody/7076)), from Cell Signaling Technology via Bionordica.

Dilution for WB: 1:3000 in 1% milk 0.1% Tween20 in TBS

**References**

1 Wiśniewski JR, Zougman A, Nagaraj N, Mann M. Universal sample preparation method for proteome analysis. Nat Methods. May 2009;6(5):359-62.

2 Nesvizhskii AI, Keller A, Kolker E, Aebersold R. A statistical model for identifying proteins by tandem mass spectrometry. Anal Chem. Sep 1 2003;75(17):4646-58.

3 Wang X, Spandidos A, Wang H, Seed B. PrimerBank: a PCR primer database for quantitative gene expression analysis, 2012 update. Nucleic Acids Res. Jan 2012;40(Database issue):D1144-9.

4 Rozen S, Skaletsky H. Primer3 on the WWW for general users and for biologist programmers. Methods Mol Biol. 2000;132:365-86.
