# Supplementary material for: Deregulation of the Kallikrein Protease Family in the Salivary Glands of the Sjögren’s Syndrome ERdj5 Knockout Mouse Model
Source: Front Immunol. 2021 Jul 7;12:693911. doi: 10.3389/fimmu.2021.693911 (PMC8292930; doi:10.3389/fimmu.2021.693911)
Supplement: Supplementary Image 1 — Classification of proteins with significant proteomic relative abundance difference in the comparisons between wildtypes and knockouts according to KEGG pathways and reactome pathways in STRING analysis. Results for both sexes are presented. The proteins which were identified with significantly different relative abundances but were not part of any of the specific pathways are not presented. [file DataSheet_1.zip › 693911_SupMaterial/Table 1.DOCX]

| **Table 1.** Identified sexual dimorphic proteins in the submandibular salivary gland of mice: proteins with significant differences in computed relative abundances between female and male wildtype animals. p values <0.05 were considered significant. | | | |
| --- | --- | --- | --- |
| **Name** | **NSAF**  p-value | **emPAI**  p-value | **♂ / ♀ ratio**  NSAF \| emPAI |
| Myosin-11 | 0.00001 | 0.00001 | 2.9 \| 4 |
| Pro-epidermal growth factor | 0.00001 | 0.00001 | 16 \| 18 |
| Kallikrein 1-related peptidase b24 | 0.00001 | 0.00001 | 7.5 \| 3.7 |
| Kallikrein 1-related peptidase b26 | 0.00001 | 0.00001 | 6.5 \| 4.5 |
| Kallikrein 1-related peptidase b27 | 0.00001 | 0.00001 | 23 \| 7.4 |
| Kallikrein 1-related peptidase b1 | 0.00001 | 0.00001 | 18 \| 5.3 |
| Kallikrein 1-related peptidase-like b4 | 0.00001 | 0.0021 | 6.6 \| 3.4 |
| Kallikrein 1-related peptidase b9 | 0.00001 | 0.00001 | 15 \| 8.6 |
| Heterogeneous nuclear ribonucleoprotein A3 | 0.00001 | 0.00001 | 0.1 \| 0.1 |
| Long-chain specific acyl-CoA dehydrogenase, mitoch. | 0.00001 | 0.00001 | 0.3 \| 0.2 |
| Long-chain-fatty-acid--CoA ligase 1 | 0.00001 | 0.00019 | 0 \| 0 |
| Mitochondrial 10-formyltetrahydrofolate dehydrogenase | 0.00001 | 0.00001 | 0 \| 0 |
| Delta-1-pyrroline-5-carboxylate dehydrogenase, mitoch. | 0.00001 | 0.0031 | 0 \| 0 |
| Aldose reductase | 0.00001 | 0.00028 | 0.1 \| 0.2 |
| Carboxypeptidase D | 0.00001 | 0.00068 | 0 \| 0 |
| Cysteine-rich protein 2 | 0.00001 | 0.00001 | 0 \| 0 |
| Fructose-1,6-bisphosphatase 1 | 0.00001 | 0.00001 | 0 \| 0 |
| Protein FAM3D | 0.00001 | 0.00026 | 0 \| 0 |
| Heat shock cognate 71 kDa protein | 0.00001 | 0.00001 | 0.5 \| 0.5 |
| cAMP-dependent protein kinase type II-beta regulatory subunit | 0.00001 | 0.00035 | 0 \| 0 |
| Kallikrein-1 | 0.00001 | 0.0012 | 0.1 \| 0.3 |
| Laminin subunit alpha-2 | 0.00001 | 0.0025 | 0.04 \| 0.05 |
| L-lactate dehydrogenase B chain | 0.00001 | 0.0078 | 0 \| 0 |
| Methylcrotonoyl-CoA carboxylase beta chain, mitoch. | 0.00001 | 0.00067 | 0 \| 0 |
| Vesicle-fusing ATPase | 0.00001 | 0.00001 | 0 \| 0 |
| Pyrroline-5-carboxylate reductase 1, mitoch. | 0.00001 | 0.00001 | 0.08 \| 0.09 |
| Prolactin-inducible protein homolog | 0.00001 | 0.00001 | 0 \| 0 |
| Protein Lpo | 0.00001 | 0.00001 | 0.08 \| 0.1 |
| 40S ribosomal protein S21 | 0.00001 | 0.00059 | 0 \| 0 |
| 40S ribosomal protein S3 | 0.00001 | 0.00001 | 0.3 \| 0.2 |
| D-3-phosphoglycerate dehydrogenase | 0.00001 | 0.00001 | 0.02 \| 0.03 |
| Staphylococcal nuclease domain-containing protein 1 | 0.00001 | 0.00012 | 0.3 \| 0.3 |
| Transmembrane 9 superfamily m. 2 | 0.00001 | 0.0061 | 0.1 \| 0.2 |
| Isof. 2 of Synaptophysin-like protein 1 | 0.00001 | 0.00001 | 0 \| 0 |
| Isof. 2 of Glutamine--fructose-6-phosphate aminotransferase [isomerizing] 1 | 0.00001 | 0.00001 | 0.05 \| 0.06 |
| Submandibular gland protein C | 0.00001 | 0.00001 | 0 \| 0 |
| LIM domain and actin-binding protein 1 | 0.00001 | 0.0017 | 0 \| 0 |
| NAD(P) transhydrogenase, mitoch. | 0.0001 | 0.0011 | 0.05 \| 0.05 |
| Plastin-2 | 0.0001 | 0.00001 | 0.1 \| 0.1 |
| CD81 antigen | 0.00011 | 0.00001 | 0 \| 0 |
| Solute carrier family 12 m. 2 | 0.00011 | 0.00054 | 0.3 \| 0.4 |
| Electron transfer flavoprotein-ubiquinone oxidoreductase, mitoch. | 0.00011 | 0.00001 | 0 \| 0 |
| Spectrin beta chain, non-erythrocytic 1 | 0.00011 | 0.0021 | 0.2 \| 0.2 |
| Ribosome-binding protein 1 | 0.00012 | 0.0032 | 0.4 \| 0.5 |
| Collagen alpha-6(VI) chain | 0.00013 | 0.00001 | 0 \| 0 |
| Centromere protein V | 0.00017 | 0.0018 | 0.2 \| 0.2 |
| Protein phosphatase 1 regulatory subunit 1B | 0.00019 | 0.00012 | 0 \| 0 |
| Stress-70 protein, mitoch. | 0.0002 | 0.0025 | 0.2 \| 0.3 |
| Aspartate aminotransferase, mitoch. | 0.00022 | 0.0017 | 0.2 \| 0.3 |
| Ubiquitin carboxyl-terminal hydrolase isozyme L3 | 0.00022 | 0.0063 | 0 \| 0 |
| Kallikrein 1-related peptidase b11 | 0.00023 | 0.0032 | 5.4 \| 3.9 |
| CD82 antigen | 0.00026 | 0.00001 | 0 \| 0 |
| UMP-CMP kinase | 0.00027 | 0.00086 | 0.2 \| 0.2 |
| Cytochrome c, somatic | 0.00028 | 0.00001 | 0.2 \| 0.2 |
| Pyruvate carboxylase | 0.00028 | 0.0003 | 0.04 \| 0.05 |
| Transmembrane protein 214 | 0.0003 | 0.00001 | 0 \| 0 |
| DDRGK domain-containing protein 1 | 0.00035 | 0.00001 | 0 \| 0 |
| CMP-N-acetylneuraminate-beta-galactosamide-alpha-2,3-sialyltransferase 4 | 0.00036 | 0.00026 | 0 \| 0 |
| Kallikrein 1-related peptidase b21 | 0.00037 | 0.00001 | 5.9 \| 6.7 |
| Elongation factor 1-gamma | 0.00038 | 0.0022 | 0.1 \| 0.1 |
| Prominin-2 | 0.00038 | 0.00001 | 0.06 \| 0.07 |
| Protein-arginine deiminase type-2 | 0.00039 | 0.0001 | 0.03 \| 0.04 |
| Laminin subunit beta-1 | 0.00041 | 0.00067 | 0.3 \| 0.4 |
| Synaptic vesicle membrane protein VAT-1 homolog | 0.00042 | 0.0011 | 0.2 \| 0.3 |
| Kallikrein 1-related peptidase b16 | 0.00044 | 0.00015 | 5.3 \| 3.9 |
| Phosphatidylethanolamine-binding protein 1 | 0.00044 | 0.0053 | 0.1 \| 0.2 |
| 40S ribosomal protein S16 | 0.00044 | 0.00025 | 0.03 \| 0.03 |
| Phosphatidylinositide phosphatase SAC1 | 0.00044 | 0.00001 | 0 \| 0 |
| Leucine--tRNA ligase, cytoplasmic | 0.00044 | 0.005 | 0.05 \| 0.04 |
| Ras-related protein Rap-1b | 0.00048 | 0.00091 | 0 \| 0 |
| Cytoplasmic dynein 1 intermediate chain 2 | 0.00055 | 0.00001 | 3 \| 4.3 |
| Signal recognition particle subunit SRP68 | 0.00056 | 0.00001 | 0 \| 0 |
| Protein ERGIC-53 | 0.00059 | 0.00051 | 0.2 \| 0.2 |
| Aldehyde dehydrogenase | 0.00061 | 0.00042 | 0.3 \| 0.4 |
| Isocitrate dehydrogenase 3 (NAD+) beta | 0.00066 | 0.0033 | 0.05 \| 0.05 |
| Coatomer subunit beta | 0.00069 | 0.025 | 0.4 \| 0.5 |
| Pyruvate dehydrogenase E1 component subunit beta, mitoch. | 0.00074 | 0.011 | 0.3 \| 0.3 |
| Heat shock protein HSP 90-beta | 0.00075 | 0.0012 | 0.2 \| 0.4 |
| Isof. 3 of Heterogeneous nuclear ribonucleoprotein D0 | 0.00078 | 0.0025 | 0.2 \| 0.08 |
| Succinyl-CoA ligase [GDP-forming] subunit beta, mitoch. | 0.00081 | 0.0017 | 0.08 \| 0.1 |
| Translocon-associated protein subunit gamma | 0.00084 | 0.0016 | 0 \| 0 |
| Threonine--tRNA ligase, cytoplasmic | 0.00085 | 0.0047 | 0.2 \| 0.2 |
| 60S ribosomal protein L5 | 0.00086 | 0.00023 | 0.2 \| 0.2 |
| T-complex protein 1 subunit beta | 0.00092 | 0.0069 | 0.2 \| 0.3 |
| Succinyl-CoA:3-ketoacid coenzyme A transferase 1, mitoch. | 0.00093 | 0.0076 | 0.04 \| 0.05 |
| Eukaryotic translation initiation factor 2 subunit 2 | 0.00098 | 0.0031 | 0.1 \| 0.2 |
| Bifunctional UDP-N-acetylglucosamine 2-epimerase/N-acetylmannosamine kinase | 0.00099 | 0.0006 | 0 \| 0 |
| Ras GTPase-activating-like protein IQGAP2 | 0.001 | 0.0011 | 0.3 \| 0.4 |
| Alpha-actinin-4 | 0.0011 | 0.00054 | 0.3 \| 0.3 |
| Histone H1.5 | 0.0011 | 0.0031 | 0.2 \| 0.07 |
| 28 kDa heat- and acid-stable phosphoprotein | 0.0011 | 0.017 | 0 \| 0 |
| Prohibitin | 0.0011 | 0.011 | 0.1 \| 0.2 |
| Heat shock 70 kDa protein 4 | 0.0011 | 0.0026 | 0.2 \| 0.3 |
| Eukaryotic translation initiation factor 4B | 0.0012 | 0.0028 | 0.1 \| 0.2 |
| Complement C3 | 0.0012 | 0.025 | 0.1 \| 0.1 |
| Kallikrein 1-related peptidase b3 | 0.0013 | 0.0002 | 5.6 \| 6.1 |
| Kallikrein 1-related peptidase b8 | 0.0013 | 0.00001 | 8.1 \| 8.4 |
| Cytochrome b-5, isoform CRA_a | 0.0013 | 0.0012 | 0.09 \| 0.09 |
| Protein RCC2 | 0.0013 | 0.011 | 0 \| 0 |
| 40S ribosomal protein SA | 0.0013 | 0.0019 | 0.3 \| 0.3 |
| Fatty aldehyde dehydrogenase | 0.0014 | 0.00001 | 0.1 \| 0.07 |
| Membrane-associated progesterone receptor component 2 | 0.0014 | 0.01 | 0 \| 0 |
| Succinate dehydrogenase [ubiquinone] flavoprotein subunit, mitoch. | 0.0014 | 0.0036 | 0.3 \| 0.4 |
| Histone H1.2 | 0.0015 | 0.012 | 0.1 \| 0.3 |
| Chloride intracellular channel protein 4 | 0.0016 | 0.0019 | 0 \| 0 |
| Trifunctional enzyme subunit beta, mitoch. | 0.0016 | 0.00041 | 0.1 \| 0.1 |
| Laminin subunit gamma-1 | 0.0016 | 0.023 | 0.3 \| 0.4 |
| Phosphoglycolate phosphatase | 0.0016 | 0.0024 | 0 \| 0 |
| Lysosomal alpha-mannosidase | 0.0017 | 0.00001 | 2.6 \| 2.9 |
| Sodium/potassium-transporting ATPase subunit alpha-1 | 0.0017 | 0.0042 | 0.4 \| 0.5 |
| Mannose-1-phosphate guanyltransferase beta | 0.0017 | 0.01 | 0.09 \| 0.1 |
| 60S ribosomal protein L24 | 0.0017 | 0.0068 | 0.1 \| 0.2 |
| Dolichyl-diphosphooligosaccharide--protein glycosyltransferase subunit 1 | 0.0017 | 0.025 | 0.3 \| 0.4 |
| Branched-chain-amino-acid aminotransferase, mitoch. | 0.0018 | 0.035 | 0.2 \| 0.4 |
| UDP-N-acetylhexosamine pyrophosphorylase | 0.0018 | 0.00001 | 0 \| 0 |
| Serine--tRNA ligase, cytoplasmic | 0.0018 | 0.00044 | 0.1 \| 0.1 |
| Selenium-binding protein 1 | 0.0018 | 0.0023 | 0 \| 0 |
| Aspartate aminotransferase, cytoplasmic | 0.0019 | 0.00011 | 0 \| 0 |
| UTP--glucose-1-phosphate uridylyltransferase | 0.0019 | 0.0081 | 0 \| 0 |
| Omega-amidase NIT2 | 0.002 | 0.014 | 0 \| 0 |
| Prolargin | 0.002 | 0.0033 | 0 \| 0 |
| Cytochrome b-c1 complex subunit 2, mitoch. | 0.002 | 0.024 | 0.5 \| 0.6 |
| C-1-tetrahydrofolate synthase, cytoplasmic | 0.0021 | 0.013 | 0.2 \| 0.2 |
| Isof. 2 of ATP-dependent (S)-NAD(P)H-hydrate dehydratase | 0.0021 | 0.0014 | 0 \| 0 |
| Eukaryotic translation initiation factor 3 subunit G | 0.0022 | 0.0081 | 0 \| 0 |
| Alanine--tRNA ligase, cytoplasmic | 0.0022 | 0.00083 | 0.07 \| 0.07 |
| Carboxylesterase 1D | 0.0024 | 0.0014 | 0.1 \| 0.2 |
| Eukaryotic translation initiation factor 3 subunit L | 0.0024 | 0.0024 | 0.09 \| 0.1 |
| Propionyl-CoA carboxylase alpha chain, mitoch. | 0.0024 | 0.032 | 0.3 \| 0.4 |
| T-complex protein 1 subunit alpha | 0.0024 | 0.01 | 0.2 \| 0.2 |
| Isof. 3 of NSFL1 cofactor p47 | 0.0024 | 0.014 | 0.2 \| 0.2 |
| 60 kDa heat shock protein, mitoch. | 0.0025 | 0.047 | 0.7 \| 0.7 |
| Coatomer subunit beta' | 0.0026 | 0.0019 | 0.3 \| 0.4 |
| Dolichyl-diphosphooligosaccharide--protein glycosyltransferase subunit DAD1 | 0.0026 | 0.0036 | 0 \| 0 |
| Electron transfer flavoprotein subunit beta | 0.0026 | 0.0089 | 0.4 \| 0.4 |
| Asparagine synthetase [glutamine-hydrolyzing] | 0.0027 | 0.00088 | 0.1 \| 0.1 |
| Isof. 2 of AP-1 complex subunit mu-2 | 0.0027 | 0.0047 | 0 \| 0 |
| Beta-nerve growth factor | 0.0029 | 0.0057 | 20 \| 4.7 |
| Actin-related protein 2 | 0.0029 | 0.023 | 0 \| 0 |
| RNA-binding protein 47 | 0.0029 | 0.015 | 0.1 \| 0.2 |
| Cytochrome c oxidase subunit 7A2, mitoch. | 0.003 | 0.0088 | 0.2 \| 0.3 |
| Serum albumin | 0.0032 | 0.016 | 0.5 \| 0.5 |
| Peptidyl-prolyl cis-trans isomerase FKBP4 | 0.0032 | 0.0035 | 0 \| 0 |
| Kinesin-1 heavy chain | 0.0033 | 0.0054 | 0.1 \| 0.1 |
| Alpha-parvin | 0.0034 | 0.0096 | 0 \| 0 |
| 40S ribosomal protein S8 | 0.0034 | 0.0067 | 0.2 \| 0.3 |
| Apoptosis-associated speck-like protein containing a CARD | 0.0036 | 0.00086 | 0.1 \| 0.2 |
| Hsc70-interacting protein | 0.0036 | 0.014 | 0.1 \| 0.2 |
| Ras-related protein Rab-3D | 0.0037 | 0.0026 | 0.03 \| 0.07 |
| Neprilysin | 0.0039 | 0.017 | 2.7 \| 3.1 |
| Prolactin regulatory element binding, isoform CRA_b | 0.0039 | 0.039 | 0 \| 0 |
| 60S ribosomal protein L7 | 0.004 | 0.023 | 0.4 \| 0.5 |
| Syndecan-4 | 0.004 | 0.0032 | 0 \| 0 |
| Heat shock 70 kDa protein 1A | 0.0041 | 0.028 | 0.2 \| 0.4 |
| Transaldolase | 0.0041 | 0.018 | 0.3 \| 0.3 |
| Ragulator complex protein LAMTOR1 | 0.0043 | 0.0016 | 9.1 \| 17 |
| Sodium/potassium-transporting ATPase subunit beta-1 | 0.0044 | 0.016 | 0.3 \| 0.4 |
| Protein Prol1 | 0.0046 | 0.00013 | 0 \| 0 |
| Protein Gm20425 | 0.0046 | 0.0029 | 0.5 \| 0.5 |
| Mitochondrial amidoxime reducing component 2 | 0.0046 | 0.033 | 0.2 \| 0.3 |
| 4-trimethylaminobutyraldehyde dehydrogenase | 0.0046 | 0.00061 | 0.09 \| 0.1 |
| Dihydropyrimidine dehydrogenase [NADP(+)] | 0.0048 | 0.004 | 0 \| 0 |
| Erlin-2 | 0.0048 | 0.0018 | 0 \| 0 |
| Fatty acid-binding protein, epidermal | 0.0052 | 0.0044 | 0.4 \| 0.3 |
| Basement membrane-specific heparan sulfate proteoglycan core protein | 0.0053 | 0.02 | 0.2 \| 0.3 |
| Serum paraoxonase/lactonase 3 | 0.0053 | 0.0013 | 0.1 \| 0.1 |
| ABPBG26 | 0.0053 | 0.024 | 0.1 \| 0.2 |
| 40S ribosomal protein S2 | 0.0054 | 0.0031 | 0.2 \| 0.2 |
| Isof. 2 of 40S ribosomal protein S24 | 0.0054 | 0.0015 | 0 \| 0 |
| Signal recognition particle subunit SRP72 | 0.0055 | 0.042 | 0.2 \| 0.3 |
| Kelch domain-containing protein 7A | 0.0058 | 0.011 | 0 \| 0 |
| Propionyl-CoA carboxylase beta chain, mitoch. | 0.006 | 0.017 | 0.2 \| 0.3 |
| 60S ribosomal protein L28 | 0.0062 | 0.0056 | 0.2 \| 0.2 |
| Myosin light chain kinase, smooth muscle | 0.0063 | 0.012 | 18 \| 19 |
| 60S ribosomal protein L6 | 0.0064 | 0.046 | 0.3 \| 0.4 |
| Isof. 2 of Pyridoxal-dependent decarboxylase domain-containing protein 1 | 0.0067 | 0.0062 | 0.1 \| 0.2 |
| Isof. 2 of Heterogeneous nuclear ribonucleoprotein K | 0.007 | 0.049 | 0.5 \| 0.6 |
| Lipopolysaccharide-responsive and beige-like anchor protein | 0.0071 | 0.005 | 5.7 \| 6.7 |
| Phosphomannomutase 2 | 0.0073 | 0.0057 | 0 \| 0 |
| Glutamine synthetase | 0.0077 | 0.014 | 0.4 \| 0.4 |
| ADP-ribosylation factor 5 | 0.0078 | 0.003 | 0 \| 0 |
| Transketolase | 0.008 | 0.03 | 0.5 \| 0.7 |
| Eukaryotic translation initiation factor 3 subunit C | 0.0081 | 0.0043 | 0.07 \| 0.09 |
| Peptidyl-prolyl cis-trans isomerase A | 0.0081 | 0.0051 | 0.3 \| 0.2 |
| Heterogeneous nuclear ribonucleoprotein F | 0.0081 | 0.0066 | 0.2 \| 0.2 |
| Filamin-B | 0.0084 | 0.012 | 0.3 \| 0.3 |
| Ig alpha chain C region | 0.0088 | 0.021 | 0.2 \| 0.4 |
| NADH dehydrogenase [ubiquinone] iron-sulfur protein 2, mitoch. | 0.0088 | 0.0037 | 0 \| 0 |
| Ornithine aminotransferase, mitoch. | 0.0089 | 0.0066 | 0.07 \| 0.09 |
| NADH dehydrogenase [ubiquinone] flavoprotein 1, mitoch. | 0.009 | 0.021 | 0.2 \| 0.2 |
| Prolyl endopeptidase | 0.0091 | 0.039 | 0.2 \| 0.3 |
| Ras-related protein Rab-1A | 0.0092 | 0.018 | 0.4 \| 0.5 |
| 40S ribosomal protein S27 | 0.0093 | 0.01 | 0.07 \| 0.06 |
| Carboxylesterase 1C | 0.0094 | 0.0024 | 0.08 \| 0.07 |
| 60S ribosomal protein L27a | 0.0096 | 0.0061 | 0 \| 0 |
| Creatine kinase B-type | 0.0097 | 0.017 | 3.2 \| 3.3 |
| ATP-citrate synthase | 0.0097 | 0.0051 | 0.2 \| 0.3 |
| Annexin | 0.01 | 0.021 | 0.5 \| 0.6 |
| 60S ribosomal protein L30 | 0.01 | 0.016 | 0.1 \| 0.2 |
| Isof. 2 of ATP-dependent 6-phosphofructokinase, muscle type | 0.011 | 0.012 | INF \| INF |
| Isof. 2 of Carboxypeptidase Q | 0.011 | 0.025 | 4 \| 3 |
| Density-regulated protein | 0.011 | 0.024 | 0.1 \| 0.1 |
| 2-amino-3-ketobutyrate coenzyme A ligase, mitoch. | 0.011 | 0.016 | 0 \| 0 |
| T-complex protein 1 subunit delta | 0.011 | 0.0034 | 0.2 \| 0.09 |
| Transmembrane 9 superfamily m. 3 | 0.011 | 0.014 | 0 \| 0 |
| 6-phosphogluconate dehydrogenase, decarboxylating | 0.012 | 0.021 | 0.3 \| 0.4 |
| Alcohol dehydrogenase class-3 | 0.012 | 0.038 | 0.2 \| 0.2 |
| ATP synthase subunit d, mitoch. | 0.012 | 0.011 | 0.4 \| 0.4 |
| Eukaryotic translation initiation factor 4 gamma 1 | 0.012 | 0.014 | 0.4 \| 0.4 |
| Vesicle-trafficking protein SEC22b | 0.012 | 0.042 | 0.4 \| 0.5 |
| Major urinary protein 5 | 0.012 | 0.041 | 0 \| 0 |
| Peroxiredoxin-1 | 0.012 | 0.011 | 0.5 \| 0.4 |
| 60S ribosomal protein L26 | 0.012 | 0.017 | 0.3 \| 0.4 |
| Proliferation-associated protein 2G4 | 0.012 | 0.0064 | 0.1 \| 0.2 |
| Cysteine-rich secretory protein 1 | 0.013 | 0.013 | INF \| INF |
| Heterogeneous nuclear ribonucleoprotein U, isoform CRA_b | 0.013 | 0.016 | 0.1 \| 0.2 |
| Vesicular integral-membrane protein VIP36 | 0.013 | 0.013 | 0 \| 0 |
| 40S ribosomal protein S4, X isoform | 0.013 | 0.026 | 0.3 \| 0.3 |
| Thimet oligopeptidase | 0.013 | 0.0037 | 0 \| 0 |
| Protein LEG1 homolog | 0.014 | 0.002 | 7.1 \| 8.4 |
| Dolichyl-phosphate beta-glucosyltransferase | 0.014 | 0.031 | 0.1 \| 0.1 |
| Protein Tmed7 | 0.014 | 0.033 | 0.2 \| 0.3 |
| Fibrinogen beta chain | 0.014 | 0.039 | 0 \| 0 |
| Ras GTPase-activating-like protein IQGAP1 | 0.014 | 0.009 | 0.3 \| 0.4 |
| Protein LYRIC | 0.014 | 0.04 | 0 \| 0 |
| Kars protein | 0.014 | 0.047 | 0 \| 0 |
| ATP-binding cassette sub-family F m. 1 | 0.015 | 0.043 | 0 \| 0 |
| Aminoacylase-1 | 0.015 | 0.043 | 0 \| 0 |
| Biliverdin reductase A | 0.015 | 0.047 | 0 \| 0 |
| Methylmalonate-semialdehyde dehydrogenase [acylating], mitoch. | 0.015 | 0.049 | 0.6 \| 0.7 |
| Serine/arginine-rich splicing factor 2 | 0.015 | 0.0084 | 0.1 \| 0.1 |
| Transmembrane emp24 domain-containing protein 9 | 0.015 | 0.0031 | 0 \| 0 |
| Isof. 2 of Acylamino-acid-releasing enzyme | 0.015 | 0.039 | 0 \| 0 |
| Prosaposin | 0.016 | 0.012 | 3.3 \| 4.6 |
| Keratin, type I cytoskeletal 10 | 0.016 | 0.0015 | 0 \| 0 |
| Aldose 1-epimerase | 0.016 | 0.017 | 0 \| 0 |
| GDP-mannose 4,6 dehydratase | 0.016 | 0.013 | 0 \| 0 |
| 40S ribosomal protein S14 | 0.016 | 0.04 | 0.4 \| 0.4 |
| Neutral alpha-glucosidase AB | 0.016 | 0.047 | 0.2 \| 0.3 |
| General vesicular transport factor p115 | 0.016 | 0.0044 | 0.4 \| 0.5 |
| Coatomer subunit delta | 0.017 | 0.03 | 0.3 \| 0.3 |
| Heterogeneous nuclear ribonucleoprotein L (Fragment) | 0.017 | 0.036 | 0.3 \| 0.3 |
| Lamin-B1 | 0.017 | 0.032 | 0 \| 0 |
| Bifunctional epoxide hydrolase 2 | 0.017 | 0.033 | 0 \| 0 |
| Syntaxin-binding protein 2 (Fragment) | 0.018 | 0.032 | 22 \| 18 |
| Calponin-1 | 0.018 | 0.0032 | 5.3 \| 4.4 |
| Cytosolic 10-formyltetrahydrofolate dehydrogenase | 0.018 | 0.035 | 0 \| 0 |
| O-acetyl-ADP-ribose deacetylase MACROD1 | 0.018 | 0.027 | 0 \| 0 |
| Polyadenylate-binding protein 1 | 0.019 | 0.0056 | 0.3 \| 0.4 |
| Phosphoglycerate mutase 1 | 0.019 | 0.022 | 0.4 \| 0.5 |
| Isof. 2 of Heterogeneous nuclear ribonucleoprotein M | 0.019 | 0.038 | 0 \| 0 |
| Cystatin-B | 0.02 | 0.038 | 0 \| 0 |
| Spermidine synthase | 0.02 | 0.021 | 0 \| 0 |
| Transmembrane 9 superfamily m. 4 | 0.02 | 0.018 | 0 \| 0 |
| Isof. 2 of 4F2 cell-surface antigen heavy chain | 0.02 | 0.017 | 0.1 \| 0.1 |
| Programmed cell death protein 4 | 0.021 | 0.04 | 0.1 \| 0.1 |
| Prothymosin alpha | 0.021 | 0.015 | 0.2 \| 0.2 |
| 40S ribosomal protein S23 | 0.021 | 0.027 | 0.3 \| 0.3 |
| N-acylneuraminate cytidylyltransferase | 0.021 | 0.026 | 0.1 \| 0.2 |
| Glutaryl-CoA dehydrogenase, mitoch. | 0.022 | 0.044 | 0 \| 0 |
| Kynurenine--oxoglutarate transaminase 1 | 0.022 | 0.024 | 0 \| 0 |
| Neurocalcin-delta | 0.022 | 0.02 | 0 \| 0 |
| Protein Serpinb9 | 0.022 | 0.011 | 0 \| 0 |
| 16.5 kDa submandibular gland glycoprotein | 0.022 | 0.016 | 0 \| 0 |
| Isof. Short of Beta-1,4-galactosyltransferase 1 | 0.022 | 0.013 | 0 \| 0 |
| Isof. 2 of Tryptophan--tRNA ligase, cytoplasmic | 0.022 | 0.031 | 0 \| 0 |
| Apoptosis facilitator Bcl-2-like protein 14 | 0.023 | 0.035 | INF \| INF |
| ATP-binding cassette sub-family E m. 1 | 0.024 | 0.005 | 0 \| 0 |
| Phosphoglucomutase-1 | 0.025 | 0.046 | 0.2 \| 0.2 |
| Proteasome subunit alpha type-3 | 0.025 | 0.02 | 0 \| 0 |
| 3-mercaptopyruvate sulfurtransferase | 0.025 | 0.015 | 0 \| 0 |
| Adenosylhomocysteinase | 0.025 | 0.02 | 0.3 \| 0.3 |
| Brain acid soluble protein 1 | 0.027 | 0.016 | 0 \| 0 |
| Coatomer subunit alpha | 0.027 | 0.032 | 0.4 \| 0.4 |
| Eukaryotic translation initiation factor 3 subunit J-A | 0.027 | 0.015 | 0 \| 0 |
| Fatty acid synthase | 0.027 | 0.022 | 0.2 \| 0.3 |
| NADH dehydrogenase [ubiquinone] 1 alpha subcomplex subunit 12 | 0.027 | 0.019 | 0.2 \| 0.2 |
| 60S ribosomal protein L34 | 0.027 | 0.02 | 0 \| 0 |
| Dynein light chain 2, cytoplasmic | 0.028 | 0.022 | 0.1 \| 0.2 |
| High mobility group nucleosome-binding domain-containing protein 5 | 0.028 | 0.014 | 0 \| 0 |
| Leukotriene A-4 hydrolase | 0.028 | 0.0089 | 0.09 \| 0.1 |
| Thioredoxin-dependent peroxide reductase, mitoch. | 0.028 | 0.049 | 0.2 \| 0.3 |
| Succinate dehydrogenase [ubiquinone] iron-sulfur subunit, mitoch. | 0.028 | 0.036 | 0.3 \| 0.4 |
| DnaJ homolog subfamily C m. 3 | 0.029 | 0.028 | 2.5 \| 2.8 |
| Cytochrome P450 2B10 | 0.029 | 0.031 | INF \| INF |
| 14-3-3 protein gamma | 0.029 | 0.0035 | 0.6 \| 0.4 |
| 14-3-3 protein theta (Fragment) | 0.029 | 0.012 | 0.5 \| 0.5 |
| Radixin | 0.03 | 0.015 | 0.4 \| 0.4 |
| 40S ribosomal protein S9 | 0.03 | 0.031 | 0.2 \| 0.3 |
| GTP:AMP phosphotransferase AK3, mitoch. | 0.031 | 0.028 | 0.4 \| 0.4 |
| NADH dehydrogenase [ubiquinone] 1 alpha subcomplex subunit 13 | 0.031 | 0.015 | 0 \| 0 |
| ADP/ATP translocase 2 | 0.032 | 0.007 | 0.3 \| 0.3 |
| Signal peptidase complex subunit 2 | 0.032 | 0.043 | 0.3 \| 0.3 |
| Catalase | 0.034 | 0.031 | 0.4 \| 0.4 |
| 40S ribosomal protein S3a | 0.034 | 0.033 | 0.3 \| 0.4 |
| Isof. 2 of Protein SET | 0.034 | 0.02 | 0 \| 0 |
| Periostin | 0.036 | 0.031 | 4 \| 3.9 |
| Ras-related protein Rab-5C | 0.036 | 0.021 | 0 \| 0 |
| Eukaryotic translation initiation factor 3 subunit B | 0.038 | 0.037 | 0.08 \| 0.1 |
| Nidogen-1 | 0.038 | 0.042 | 0.2 \| 0.3 |
| Ras-related protein Rab-14 | 0.038 | 0.021 | 0.4 \| 0.4 |
| GTP-binding protein SAR1b | 0.038 | 0.028 | 0.2 \| 0.2 |
| Nucleolin | 0.039 | 0.035 | 0.2 \| 0.2 |
| 60S ribosomal protein L4 | 0.039 | 0.042 | 0.4 \| 0.5 |
| Isof. 4 of Coatomer subunit gamma-2 | 0.04 | 0.013 | 0.1 \| 0.3 |
| Cysteine-rich secretory protein 3 | 0.041 | 0.0042 | 2 \| 3.1 |
| Endoplasmic reticulum-Golgi intermediate compartment protein 1 | 0.041 | 0.021 | 0 \| 0 |
| Serine/threonine-protein phosphatase 2A catalytic subunit alpha isoform | 0.042 | 0.03 | 0 \| 0 |
| Cysteine and glycine-rich protein 1 | 0.043 | 0.022 | 1.5 \| 1.6 |
| Protein S100-A1 | 0.043 | 0.047 | 12 \| 8.3 |
| Sarcoplasmic/endoplasmic reticulum calcium ATPase 2 | 0.043 | 0.0037 | 0.5 \| 0.7 |
| Beta-hexosaminidase subunit beta | 0.044 | 0.0026 | 2.1 \| 2.7 |
| Kallikrein 1-related peptidase b22 | 0.045 | 0.041 | 5.7 \| 8.5 |
| Receptor accessory protein 5, isoform CRA_a | 0.046 | 0.041 | 0.3 \| 0.4 |
| Pyruvate dehydrogenase E1 component subunit alpha, somatic form, mitoch. | 0.049 | 0.043 | 0.2 \| 0.3 |
| Myosin-9 | 0.0008 | 0.54 | 0.4 \| 0.9 |
| Histone H1.3 | 0.001 | 0.054 | 0.6 \| 0.6 |
| Peroxiredoxin-2 | 0.0015 | 0.11 | 0.4 \| 0.7 |
| Vigilin | 0.0021 | 0.051 | 0.4 \| 0.5 |
| Malate dehydrogenase, cytoplasmic | 0.0035 | 0.5 | 0.5 \| 0.9 |
| Cytochrome c oxidase subunit 5B, mitoch. | 0.0036 | 0.088 | 0 \| 0 |
| NAD(P)H-hydrate epimerase | 0.005 | 0.081 | 0.3 \| 0.5 |
| Glutamate dehydrogenase 1, mitoch. | 0.0054 | 0.093 | 0.3 \| 0.5 |
| Glutathione S-transferase Mu 1 | 0.0054 | 0.06 | 0.3 \| 0.5 |
| Protein disulfide-isomerase | 0.0063 | 0.29 | 0.5 \| 0.8 |
| Transmembrane emp24 domain-containing protein 10 | 0.0067 | 0.059 | 0.3 \| 0.6 |
| Prelamin-A/C | 0.0075 | 0.058 | 0.5 \| 0.6 |
| Transitional endoplasmic reticulum ATPase | 0.0092 | 0.086 | 0.6 \| 0.7 |
| Spectrin alpha chain, non-erythrocytic 1 | 0.01 | 0.091 | 0.5 \| 0.7 |
| 14-3-3 protein epsilon | 0.012 | 0.087 | 0.5 \| 0.7 |
| Guanine nucleotide-binding protein subunit beta-2-like 1 | 0.012 | 0.15 | 0.5 \| 0.7 |
| Inositol monophosphatase 1 | 0.013 | 0.061 | 0 \| 0 |
| Protein Tfg | 0.013 | 0.061 | 0.3 \| 0.3 |
| Phosphoserine aminotransferase | 0.013 | 0.06 | 0 \| 0 |
| Actin, alpha cardiac muscle 1 | 0.014 | 0.079 | 1.7 \| 1.5 |
| ATP synthase subunit beta, mitoch. | 0.014 | 0.073 | 0.7 \| 0.7 |
| Mast cell carboxypeptidase A | 0.014 | 0.11 | 0.2 \| 0.3 |
| Phosphoenolpyruvate carboxykinase [GTP], mitoch. | 0.014 | 0.13 | 0.3 \| 0.5 |
| Catenin alpha-1 | 0.015 | 0.05 | 0.4 \| 0.5 |
| Alpha-enolase | 0.015 | 0.34 | 0.4 \| 0.9 |
| Isocitrate dehydrogenase [NADP] cytoplasmic | 0.015 | 0.073 | 0.3 \| 0.4 |
| Eukaryotic initiation factor 4A-I | 0.015 | 0.061 | 0.4 \| 0.5 |
| Isof. 2 of Plasminogen activator inhibitor 1 RNA-binding protein | 0.015 | 0.099 | 0.3 \| 0.4 |
| Calnexin | 0.016 | 0.22 | 0.4 \| 0.6 |
| ABPBG27 | 0.019 | 0.13 | 0.09 \| 0.4 |
| Translationally-controlled tumor protein | 0.019 | 0.094 | 0.4 \| 0.5 |
| Cytochrome c1, heme protein, mitoch. | 0.02 | 0.06 | 0.4 \| 0.5 |
| Epithelial cell adhesion molecule | 0.021 | 0.66 | 0.6 \| 0.9 |
| Keratin, type II cytoskeletal 8 | 0.021 | 0.086 | 0.7 \| 0.8 |
| Keratin, type II cytoskeletal 5 | 0.022 | 0.47 | 0.3 \| 0.7 |
| 40S ribosomal protein S11 | 0.023 | 0.59 | 0.3 \| 0.8 |
| Isof. 1 of Core histone macro-H2A.1 | 0.024 | 0.091 | 0.4 \| 0.5 |
| Hemoglobin subunit beta-2 | 0.026 | 0.83 | 0.5 \| 1.1 |
| Proteasome subunit alpha type-1 | 0.026 | 0.056 | 0.2 \| 0.2 |
| Signal peptidase complex subunit 3 | 0.026 | 0.068 | 0.3 \| 0.4 |
| Carbonic anhydrase 6 | 0.028 | 0.13 | 0.3 \| 0.6 |
| Isof. 2 of Protein HID1 | 0.028 | 0.11 | 0 \| 0 |
| NADH dehydrogenase [ubiquinone] flavoprotein 2, mitoch. | 0.029 | 0.066 | 0.2 \| 0.3 |
| Oxidation resistance protein 1 | 0.03 | 0.1 | 0.3 \| 0.3 |
| Heterogeneous nuclear ribonucleoprotein A/B | 0.03 | 0.073 | 0.4 \| 0.4 |
| Alpha-soluble NSF attachment protein | 0.03 | 0.14 | 0.3 \| 0.4 |
| Transmembrane emp24 domain-containing protein 3 | 0.03 | 0.062 | 0.1 \| 0.2 |
| Serpin B6 | 0.031 | 0.053 | 5.2 \| 6.5 |
| Calmodulin | 0.031 | 0.065 | 0.5 \| 0.6 |
| 3'(2'),5'-bisphosphate nucleotidase 1 | 0.033 | 0.073 | 0.3 \| 0.4 |
| Platelet glycoprotein 4 | 0.033 | 0.056 | 0 \| 0 |
| Endoplasmic reticulum resident protein 44 | 0.033 | 0.079 | 0.5 \| 0.6 |
| Redox-regulatory protein FAM213A | 0.033 | 0.25 | 0.4 \| 0.5 |
| Ubiquitin-like modifier-activating enzyme 5 | 0.033 | 0.076 | 0.2 \| 0.5 |
| 3-hydroxyisobutyryl-CoA hydrolase, mitoch. | 0.034 | 0.15 | 0.4 \| 0.5 |
| Phosphoglycerate kinase 1 | 0.034 | 0.7 | 0.7 \| 0.9 |
| Protein deglycase DJ-1 | 0.036 | 0.092 | 0.4 \| 0.5 |
| Cytosolic non-specific dipeptidase | 0.037 | 0.063 | 0.2 \| 0.2 |
| Carnitine O-palmitoyltransferase 1, liver isoform | 0.037 | 0.054 | 0 \| 0 |
| Guanine nucleotide-binding protein G(I)/G(S)/G(T) subunit beta-2 | 0.037 | 0.15 | 0.5 \| 0.7 |
| Proteasome subunit alpha type-5 | 0.037 | 0.16 | 0.3 \| 0.4 |
| Very long-chain specific acyl-CoA dehydrogenase, mitoch. | 0.038 | 0.1 | 0.4 \| 0.4 |
| Signal recognition particle 19 kDa protein | 0.039 | 0.055 | 0.2 \| 0.2 |
| WD repeat-containing protein 1 | 0.039 | 0.11 | 0.3 \| 0.4 |
| Laminin subunit beta-2 | 0.04 | 0.089 | 0.3 \| 0.4 |
| Serpin H1 | 0.042 | 0.053 | 9 \| 8.4 |
| GTP-binding nuclear protein Ran | 0.042 | 0.057 | 0.3 \| 0.3 |
| Dihydrolipoyllysine-residue acetyltransferase component of pyruvate dehydrogenase complex, mitoch. | 0.043 | 0.087 | 0.4 \| 0.4 |
| 60S ribosomal protein L31 | 0.044 | 0.14 | 0.4 \| 0.5 |
| ATP synthase subunit alpha, mitoch. | 0.045 | 0.44 | 0.6 \| 0.9 |
| Biglycan | 0.046 | 0.051 | 5.6 \| 6.5 |
| ATP synthase subunit gamma, mitoch. | 0.046 | 0.15 | 0.4 \| 0.5 |
| 60S ribosomal protein L17 | 0.048 | 0.26 | 0.4 \| 0.6 |
| Isof. 2 of Tropomyosin beta chain | 0.049 | 0.06 | 3.4 \| 2.7 |
| Glycine N-methyltransferase | 0.049 | 0.094 | 0 \| 0 |
| Proteasome subunit alpha type-7 | 0.049 | 0.12 | 0.2 \| 0.3 |
| Dolichyl-diphosphooligosaccharide--protein glycosyltransferase subunit STT3A | 0.049 | 0.052 | 0 \| 0 |
| Hypoxia up-regulated protein 1 | 0.059 | 0.00075 | 1.5 \| 2.6 |
| Serpin B11 | 0.063 | 0.002 | 63 \| 45 |
| Hemopexin | 0.53 | 0.002 | 1.3 \| 3 |
| Leukocyte elastase inhibitor A | 0.054 | 0.0057 | 2.6 \| 2.4 |
| Trifunctional enzyme subunit alpha, mitoch. | 0.1 | 0.0057 | 1.6 \| 2 |
| Renin-2 | 0.066 | 0.0075 | 1.9 \| 1.9 |
| EH domain-containing protein 2 | 0.051 | 0.0098 | 0.05 \| 0.08 |
| Serine protease inhibitor A3K | 0.22 | 0.014 | 2.2 \| 4.7 |
| Lumican | 0.34 | 0.015 | 1.3 \| 2.1 |
| Guanine nucleotide-binding protein G(I)/G(S)/G(T) subunit beta-1 | 0.42 | 0.015 | 0.6 \| 0.3 |
| Cytoplasmic dynein 1 heavy chain 1 | 0.17 | 0.016 | 1.3 \| 1.6 |
| Cytochrome b-c1 complex subunit Rieske, mitoch. | 0.087 | 0.018 | 0.4 \| 0.2 |
| Calcium-binding mitoch. carrier protein Aralar1 | 0.18 | 0.019 | 1.4 \| 1.7 |
| Uncharacterized protein | 0.34 | 0.023 | 1.4 \| 2.5 |
| Epoxide hydrolase 1 | 0.055 | 0.024 | 0 \| 0 |
| Alpha actinin 1a | 0.68 | 0.024 | 0.8 \| 0.4 |
| Neutral amino acid transporter ASCT2 | 0.058 | 0.026 | 0 \| 0 |
| Talin-1 | 0.36 | 0.029 | 1.2 \| 1.5 |
| Filamin, alpha | 0.093 | 0.036 | 2 \| 2.5 |
| Golgi reassembly-stacking protein 2 | 0.075 | 0.037 | 0.3 \| 0.2 |
| Annexin A6 | 0.48 | 0.037 | 0.6 \| 0.2 |
| Translocon-associated protein subunit alpha | 0.063 | 0.038 | 0.3 \| 0.3 |
| Isof. 2 of Alpha-aminoadipic semialdehyde dehydrogenase | 0.054 | 0.041 | 0.5 \| 0.5 |
| Tropomyosin alpha-1 chain | 0.057 | 0.045 | 1.5 \| 2 |
