# Supplementary material for: Deregulation of the Kallikrein Protease Family in the Salivary Glands of the Sjögren’s Syndrome ERdj5 Knockout Mouse Model
Source: Front Immunol. 2021 Jul 7;12:693911. doi: 10.3389/fimmu.2021.693911 (PMC8292930; doi:10.3389/fimmu.2021.693911)
Supplement: Supplementary Image 1 — Classification of proteins with significant proteomic relative abundance difference in the comparisons between wildtypes and knockouts according to KEGG pathways and reactome pathways in STRING analysis. Results for both sexes are presented. The proteins which were identified with significantly different relative abundances but were not part of any of the specific pathways are not presented. [file DataSheet_1.zip › 693911_SupMaterial/Table 2.DOCX]

| **Table 2.** Innate and adaptive immunity related KEGG pathways / reactome pathways that were found significantly enriched in the differentially expressed proteins in this study. | | | | | |
| --- | --- | --- | --- | --- | --- |
| **Pathway** | | **Protein** | **Gene** | **Differential quantification** | |
|  |  |  |  | In Males | In Females |
| Inflammatory mediator regulation of TRP channels | | Phospholipase C, beta 4 | Plcb4 | KO high | n/s |
|  |  | Serine/threonine-protein phosphatase PP1-alpha catalytic subunit | Ppp1ca | KO high | n/s |
|  |  | Beta-nerve growth factor | Ngf | KO low | n/s |
|  |  | Kallikrein 1-related peptidase b4 | Klk1b4 | KO low | n/s |
|  |  | Calmodulin-1 | Calm1 | n/s | KO low |
| Antigen processing and presentation |  | H-2 class I histocompatibility antigen | H2-K1 | n/s | KO high |
|  | IL-17 signaling pathway | Heat shock cognate 71 kDa protein | Hspa8 | KO low | KO low |
|  |  | Heat shock protein HSP 90-alpha | Hsp90aa1 | n/s | KO high |
|  |  | Heat shock protein HSP 90-beta | Hsp90ab1 | n/s | KO high |
|  |  | Endoplasmin | Hsp90b1 | n/s | KO low |
| Innate Immune System |  | Glycogen phosphorylase, brain form | Pygb | n/s | KO high |
|  |  | Cysteine-rich secretory protein 1 | Crisp1 | KO low | n/s |
|  | Neutrophile degranulation | Neprilysin | Mme | KO low | KO low |
|  |  | DnaJ homolog subfamily C member 3 | Dnajc3 | KO low | n/s |
|  |  | Adenylyl cyclase-associated protein 1 | Cap1 | n/s | KO high |
