# Supplementary figures and images for: Deregulation of the Kallikrein Protease Family in the Salivary Glands of the Sjögren’s Syndrome ERdj5 Knockout Mouse Model
Source: Front Immunol. 2021 Jul 7;12:693911. doi: 10.3389/fimmu.2021.693911 (PMC8292930; doi:10.3389/fimmu.2021.693911)

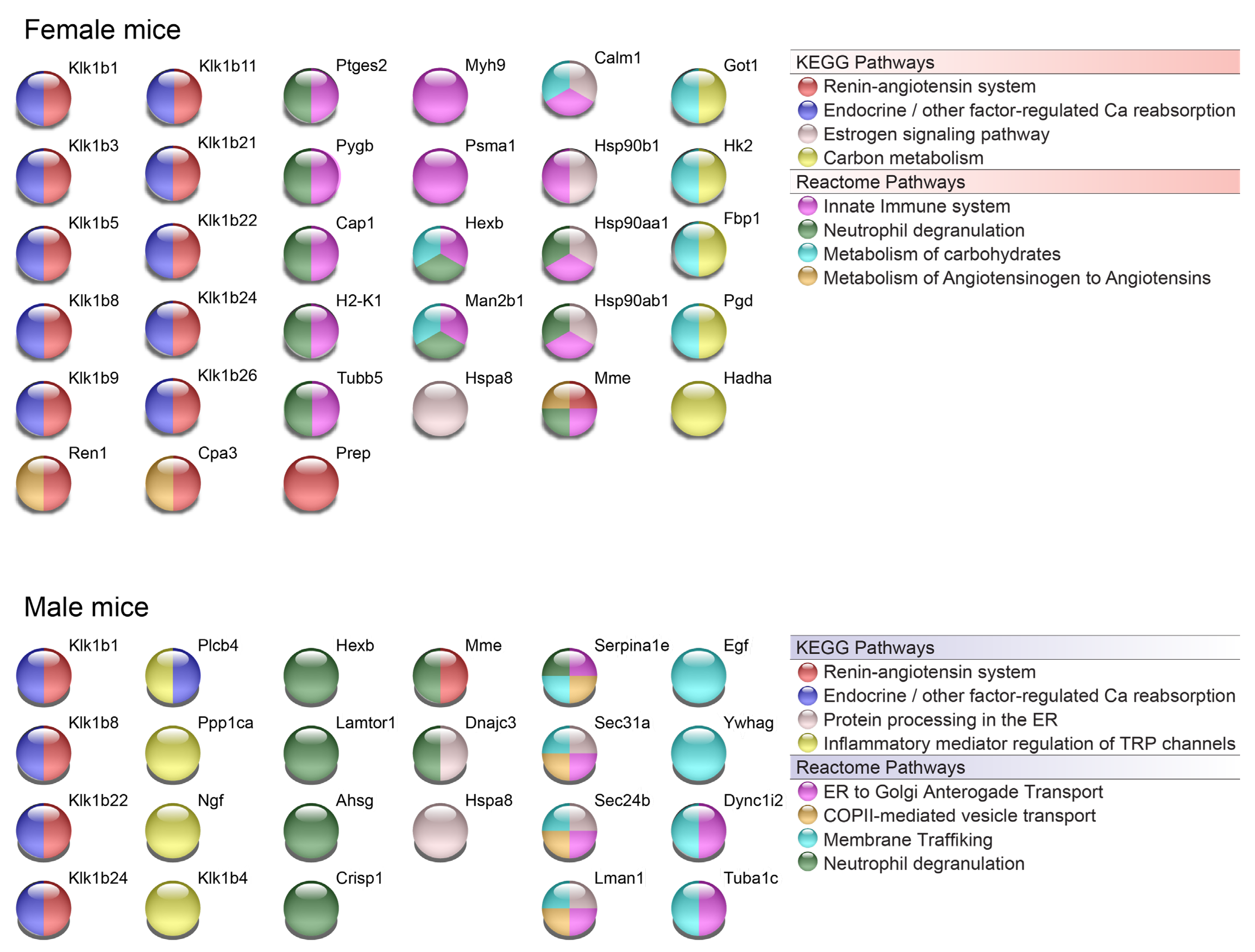

Supplement: Supplementary Image 1 — Classification of proteins with significant proteomic relative abundance difference in the comparisons between wildtypes and knockouts according to KEGG pathways and reactome pathways in STRING analysis. Results for both sexes are presented. The proteins which were identified with significantly different relative abundances but were not part of any of the specific pathways are not presented. [file DataSheet_1.zip › 693911_SupMaterial/Image 1.TIF]

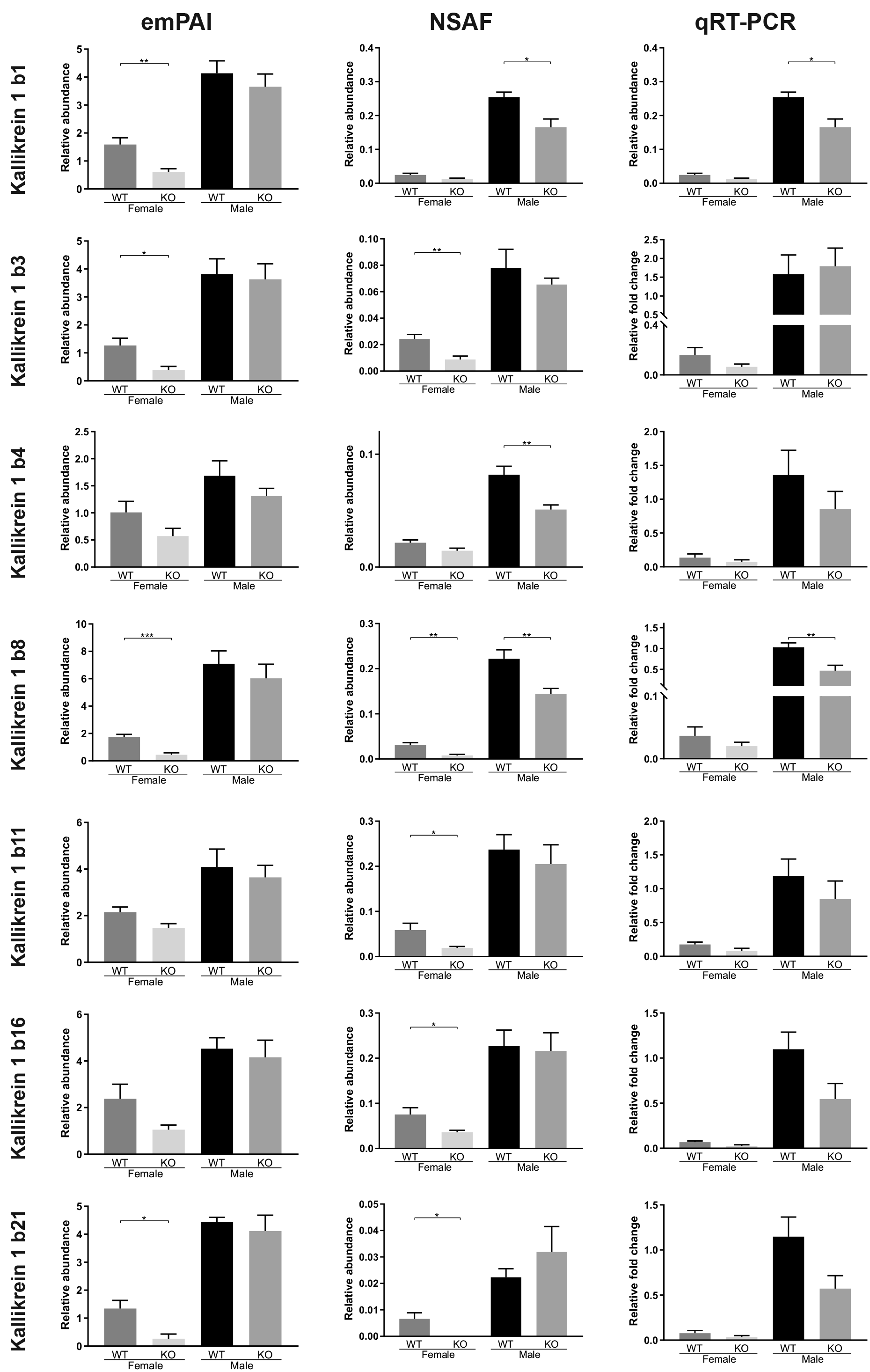

Supplement: Supplementary Image 1 — Classification of proteins with significant proteomic relative abundance difference in the comparisons between wildtypes and knockouts according to KEGG pathways and reactome pathways in STRING analysis. Results for both sexes are presented. The proteins which were identified with significantly different relative abundances but were not part of any of the specific pathways are not presented. [file DataSheet_1.zip › 693911_SupMaterial/Image 2.TIF]
